# DeepVelo: deep learning extends RNA velocity to multi-lineage systems with cell-specific kinetics

**DOI:** 10.1186/s13059-023-03148-9

**Published:** 2024-01-19

**Authors:** Haotian Cui, Hassaan Maan, Maria C. Vladoiu, Jiao Zhang, Michael D. Taylor, Bo Wang

**Affiliations:** 1 Peter Munk Cardiac Center, University Health Network, Toronto, Ontario Canada; 2 Department of Computer Science, University of Toronto, Toronto, Ontario Canada; 3 Vector Institute, Toronto, Ontario Canada; 4 Department of Medical Biophysics, University of Toronto, Toronto, Ontario Canada; 5 Department of Pathology and Molecular Medicine, McMaster University, Hamilton, Ontario Canada; 6 The Arthur and Sonia Labatt Brain Tumor Research Centre, The Hospital for Sick Children, Toronto, Ontario Canada; 7 Developmental and Stem Cell Biology Program, The Hospital for Sick Children, Toronto, Ontario Canada; 8 Baylor College of Medicine, Houston, TX USA; 9 Texas Children’s Hospital, Houston, TX USA; 10 Department of Laboratory Medicine and Pathobiology, University of Toronto, Toronto, Ontario Canada

**Keywords:** RNA velocity, Single-cell RNA sequencing, Deep Learning, Development, Cancer

## Abstract

Existing RNA velocity estimation methods strongly rely on predefined dynamics and cell-agnostic constant transcriptional kinetic rates, assumptions often violated in complex and heterogeneous single-cell RNA sequencing (scRNA-seq) data. Using a graph convolution network, DeepVelo overcomes these limitations by generalizing RNA velocity to cell populations containing time-dependent kinetics and multiple lineages. DeepVelo infers time-varying cellular rates of transcription, splicing, and degradation, recovers each cell’s stage in the differentiation process, and detects functionally relevant driver genes regulating these processes. Application to various developmental and pathogenic processes demonstrates DeepVelo’s capacity to study complex differentiation and lineage decision events in heterogeneous scRNA-seq data.

**Supplementary Information:**

The online version contains supplementary material available at 10.1186/s13059-023-03148-9.

## Background

The concept of RNA velocity refers to the time derivative of the mRNA abundance in a cell, which reflects the changing rate of RNA processing and degradation. Current velocity estimation methods leverage the observation that the abundance and ratio between unspliced pre-messenger RNAs and spliced mature messenger RNAs can be used to infer changes in gene expression dynamics. Higher abundance and ratio of unspliced mRNAs to spliced mRNAs indicates increasing transcription of a certain gene - in other words, upregulation/induction and a high velocity estimate. Conversely, a lower abundance and indicated ratio lead to a low velocity estimate associated with down-regulation/repression. An equilibrium phase occurs when this dynamic process reaches a stable steady-state. Since unspliced mRNAs can be distinguished in common single-cell RNA sequencing (scRNA-seq) protocols [1], the idea of estimating dynamic RNA velocity using only static sequencing libraries becomes feasible.

The original RNA velocity approach [1] utilized the assumption that the observed transcriptional phases in scRNA-seq last long enough to reach both an apex of induction and a quiescent steady-state equilibrium. This technique infers a per-gene *steady-state ratio* using linear regression, and then RNA velocities are calculated as the deviation of the observed ratio from the steady-state level. This workflow implies two underlying assumptions, (1) the assumption of steady-state: for every gene, sufficient number of sequenced cells are at the steady states; (2) the assumption of cell-agnostic kinetic rates: the degradation and splicing rate for each gene is shared across all cells. These assumptions are often violated in complex biological systems and bring about limitations in downstream applications, particularly when cell states are partially observed or undergo transcription dynamics more complex than the steady-state pattern. Although a later approach, scVelo [2], attempted to generalize the *steady-state* assumption by replacing these states with *four transcriptional states* and modeling them with a dynamical model, the aforementioned second limitation still remains.

Furthermore, scVelo assumes a cyclic trajectory within the four transcriptional states for all observed genes, but this assumption also rarely holds in real-world single-cell datasets with complex differentiation trajectories and multifactorial kinetics [3]. Although several related works have been further developed, including MultiVelo [4], Chromatin Velocity [5], protaccel [6] for extending Velocity beyond RNA, VeloAE [7] for denoising velocity with Deep Neural Nets, and Dynamo [8] for exploiting the metabolic labeling sequencing data, the core velocity computation follows the original ideas and therefore the aforementioned limitations still hold.

Overall, existing techniques assume each gene follows a pre-defined trajectory depicted by constant cell-agnostic kinetic rates. This workflow implies that each gene goes through the same velocity trajectory across all cell-types, and limits the application in complex cell systems. To resolve these limitations, we highlight the need for *cell-specific kinetics* which enables the modeling of multi-lineage systems with heterogeneous cell populations. We propose DeepVelo, a deep neural network based method for RNA velocity estimation. (1) DeepVelo is optimized using a newly introduced continuity framework, resulting in an approach that is unbiased from pre-defined kinetic patterns. (2) Empowered by graph convolutional networks (GCN), DeepVelo infers gene-specific and cell-specific RNA splicing and degradation rates. Therefore, compared with the cell-agnostic parameters used in existing techniques [1, 2], DeepVelo is able to model RNA velocity for differentiation dynamics of high complexity, particularly for cell populations with heterogeneous cell-types and multiple lineages.

We demonstrate the efficacy of DeepVelo on a variety of developmental and pathological scRNA-seq datasets, including dentate gyrus neurogenesis [9], pancreatic endocrinogenesis [10], hindbrain development [11], mesenchymal/chondrocyte organogenesis [12], mouse gastrulation [13], and cerebellar pilocytic astrocytoma [11]. DeepVelo yields more consistent velocity estimates and accurately identifies transcriptional states than existing models. The method further helps identify putative driver genes of these transcriptional changes, which are more likely to characterize and be involved in dictating lineage fate-decisions. The resulting velocities and driver gene analysis, on one hand, accurately recover known differentiation trajectories in challenging scenarios of time-dependent and multi-trajectory gene regulation dynamics and, on the other hand, discover novel biological insights in challenging scenarios such as pilocytic astrocytoma tumor heterogeneity. Based on these findings, we envision DeepVelo to be a useful tool for discovering functional programs and temporal dynamics using scRNA-seq data of complex biological systems.

## Results

### The DeepVelo model

**Fig. 1:**
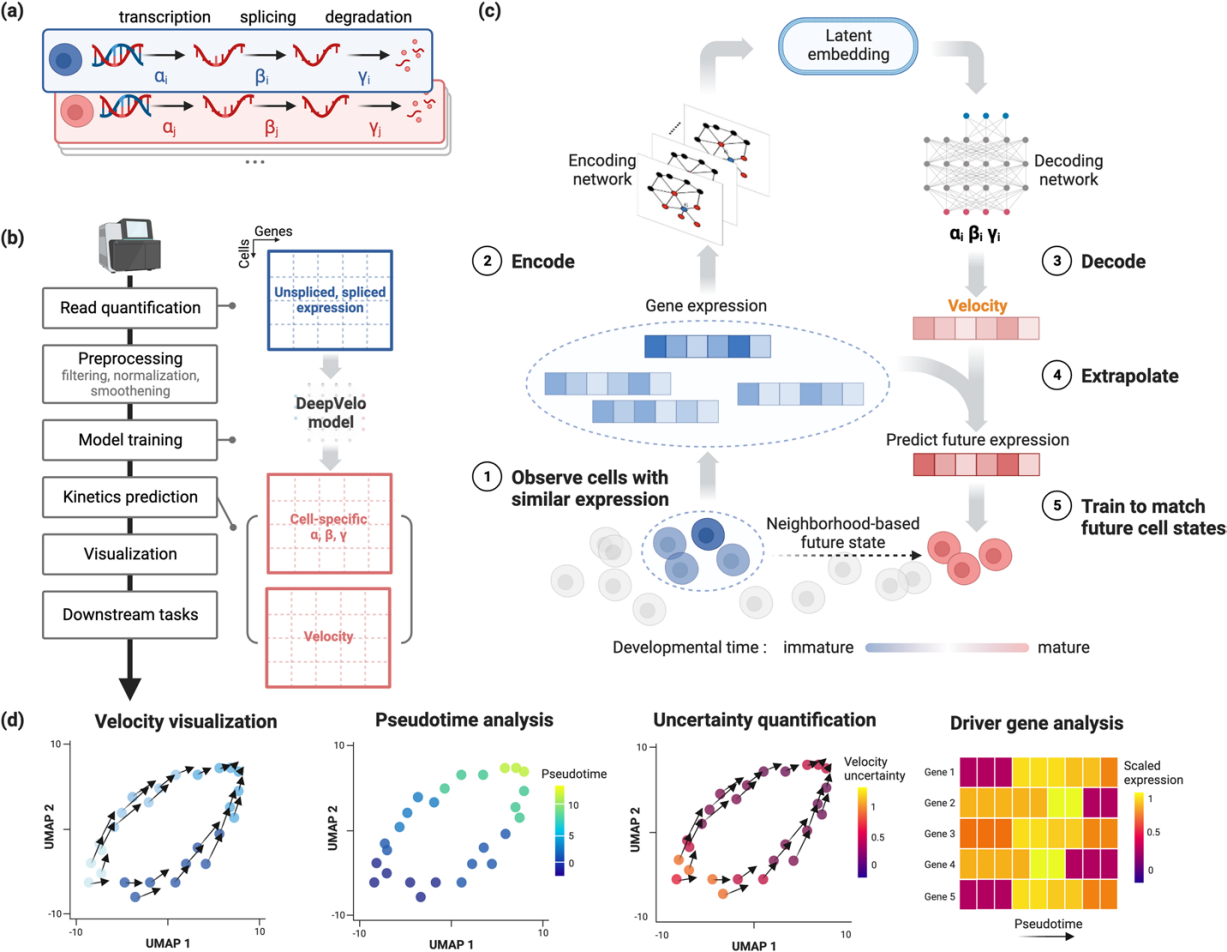
Overview of the DeepVelo pipeline and velocity prediction method. **a** DeepVelo estimates cell-specific transcription ($\alpha _i$), RNA splicing ($\beta _i$) and RNA degradation rates ($\gamma _i$). **b** Overview of the velocity analysis pipeline using DeepVelo. Preprocessing is done to ensure the stability of model training (“Continuity assumption and learning objectives”), followed by training and prediction of cell-specific kinetic parameters. These are used to estimate the RNA velocity and perform various downstream analyses. **c** Overview of the DeepVelo neural network model. Query cells (dark blue) and similar cells (light blue) within a k-nearest neighborhood are input into the model. The graph convolutional network (GCN) encoder module encodes their spliced/unspliced gene expression into latent space representations. The decoder module then predicts the kinetic rates for RNA velocity and extrapolates gene expression to future cell states. The model is optimized to match the extrapolation to observed cell states at later developmental stages. After training and optimization, these cell-specific rates can be used to determine the RNA velocity vector for each cell. **d** Downstream analyses can be performed with the DeepVelo estimated velocity results, including visualization of estimates, pseudotime analysis, assessing the confidence of velocity estimates, and selecting driver genes that are associated with the inferred development trends

Modeling the transcriptional dynamics in single cells provides the theoretical basis of RNA velocity. For each cell, the dynamics of transcription, splicing, and degradation (Fig. 1a) can be approximated as the following differential processes

1 $$\begin{aligned} \frac{{du\left( t \right) }}{{dt}} ={} & {} {\alpha _{i,g}\left( t \right) - \beta _{i,g}\left( t \right) u\left( t \right) ,} \nonumber \\ \frac{{ds\left( t \right) }}{{dt}} ={} & {} {\beta _{i,g}\left( t \right) u\left( t \right) - \gamma _{i,g}\left( t \right) s\left( t \right) .} \end{aligned}$$

where $\alpha _{i,g}, \beta _{i,g}, \gamma _{i,g}$ are the kinetic rates for cell *i* and gene *g*. *t* denotes a time coordinate in cell development. Unspliced immature mRNA is first generated by transcription of DNA and then post-transcriptionally modified and spliced into mature RNA. The dynamics of unspliced RNA abundance, $\frac{{du\left( t \right) }}{{dt}}$, is modeled by the first equation where $\alpha _{i,g}$ and $\beta _{i,g}$ denote the rates of transcription and splicing, respectively. Similarly, the second equation models the dynamics of spliced RNA abundance, $\frac{{ds\left( t \right) }}{{dt}}$, and $\gamma _{i,g}$ denotes the rate for RNA degradation. The kinetic rates are intrinsically cell-specific since there is a high degree of variability in transcriptional dynamics between cells [14]. Furthermore, these intrinsic cell-specific transcriptional dynamics are likely to be similar among similar cell-types [15], necessitating cell-type-specific parameters. However, previous velocity estimation techniques [1, 2] assume global constant kinetic rates across cells, leading to limitations in inferring multi-lineage dynamics.

DeepVelo models the kinetic rates per cell and per gene (Fig. 1a), providing sufficient expressive power for more faithful velocity estimates for individual cells. Given the unspliced gene counts *u*(*t*) and spliced gene counts *s*(*t*) for individual cells, DeepVelo estimates the derivatives of *s*(*t*) by modeling cell and gene-specific coefficients $\alpha _{i,g}, \beta _{i,g}, \gamma _{i,g}$ using a deep neural network model (Fig. 1b, c). Specifically, we predict a cell’s velocity vector and extrapolate the cell state to match the future states extracted from the sequencing data (Fig. 1c). For each cell *i* in the population, we extract a group of neighbor cells $\mathscr {N}_i$ that have similar expression profiles. We take the profiles of cell *i* and neighbor set $\mathscr {N}_i$ as the input to DeepVelo model. The model consists of stacked layers of GCNs and outputs the coefficients $\alpha _{i,g}$, $\beta _{i,g}$, and $\gamma _{i,g}$ in the final layer. Using these coefficients, DeepVelo computes the velocity $v_{i,g} = \frac{{ds\left( t \right) }}{{dt}}$ for each cell accordingly as in Eq. 1.

To train the DeepVelo model, i.e., to update the parameters for accurate velocity prediction, we first extrapolate the cell state by adding the velocity derivative $\frac{{ds\left( t \right) }}{{dt}}$ onto the original profile $s\left( t \right)$. Then, DeepVelo computes the difference between the extrapolated state $s( t+1 )$ and the real profiles of a group of downstream cells (The red cells in Fig. 1c). The DeepVelo model parameters are optimized to minimize this difference between the predicted future state and the actually observed ones (“Continuity assumption and learning objectives”). After sufficient training iterations, the model is finalized to provide accurate velocity estimates that take into account the transcriptional dynamics unique to individual cells. Notably, the above training process works in a self-supervised manner and is unbiased from cell-type annotations. The full details of the objective function of the DeepVelo model and theoretical contributions are outlined in the “Continuity assumption and learning objectives” section and Additional file 1: Note S2.

We tested DeepVelo on a number of developmental and pathological datasets to determine RNA velocity, estimate cell-specific RNA kinetics, infer developmental pseudotime, and prioritize genes for their potential role in differentiation through driver gene estimation (Fig. 1d). DeepVelo had the highest direction accuracy and consistency compared to previous approaches on almost all datasets (Additional file 1: Figs. S1, S2 and Additional file 5: Table S4).

Aside from the steady-state (Velocyto) and dynamical (scVelo) models, we further considered a recent approach that also considers multi-faceted kinetics and multi-lineage inference in cellDancer [16]. We found that cellDancer readily performed the worst out of all four methods in terms of direction score, especially on the multi-lineage hindbrain and chondrocyte datasets (Additional file 1: Fig. S1). Although the performance of cellDancer was high in terms of consistency, poor direction estimates - for example in the mouse gastrulation data with known genes that have multiple kinetics (Additional file 1: Fig. S29, Additional file 1: Note S3) - indicate that the technique does not truly resolve the problem of modeling multi-faceted kinetics and multi-lineage systems.

We also found most datasets contain a high ratio of multifaceted gene dynamics (Additional file 1: Fig. S3), and DeepVelo shows even larger margin of improvement on the most challenging ones, demonstrating the necessity and advantage of the cell-specific modeling.

### Recovering complex transcriptional dynamics for individual cells using DeepVelo

To test the ability to identify complex kinetics, we applied DeepVelo on a neurogenesis scRNA-seq dataset of the developing mouse dentate gyrus [9]. The data consists of tissue samples from two experimental time points, P12 and P35 (postnatal day 12 and 35) (Additional file 1: Fig. S4), collected by a droplet-based single-cell RNA sequencing protocol (10x Genomics Chromium Single-Cell Kit V1).

**Fig. 2:**
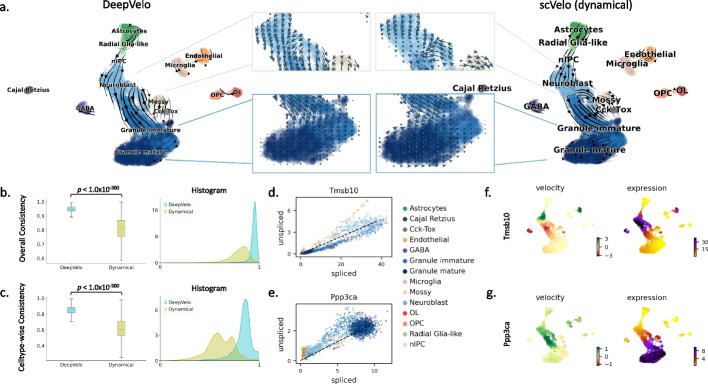
Fine-grained temporal patterns in neurogenesis predicted by DeepVelo. **a** Comparison of DeepVelo with the dynamical model from scVelo [2]. The direction and magnitude of velocities are projected as arrows onto the Uniform Manifold Approximation and Projection (UMAP) plot of gene expression values across cells. DeepVelo provides more consistent velocity estimates with respect to the developmental process from immature granule cells to mature granule cells. **b** The boxplot and histogram of the overall consistency scores for scVelo and DeepVelo, which indicate the consistency of velocity estimates in a local neighborhood of the data. **c** The box plot and histogram of the cluster/cell-type-specific consistency scores, which utilize the neighborhood consistency metric on a per cluster/cell-type basis. **d**, **e** The spliced/unspliced phase portrait for *Tmsb10* and *Ppp3ca*, respectively. Cell-types are shown in the same color as in **b**. **f**, **g** Velocity and gene expression values projected onto UMAP plots for *Tmsb10* and *Ppp3ca*, respectively. Velocity and gene expression values show consistent patterns across cell-types: high velocity values (green in velocity plot) are correctly shown in the subset of cells developing to high gene expression values (purple in expression plot)

After pre-processing (“Preprocessing the scRNA-seq data for DeepVelo”), we calculated the RNA velocities using the proposed DeepVelo model and the dynamical model from scVelo [2]. The velocity plots are made by projecting the velocity vectors onto the UMAP [17]-based embedding of the data. In the velocity estimates (Fig. 2a), the granule cell lineage dominates the main structure, where the neuroblast cells develop into immature and mature granule cells. The directions of these velocity estimates between cell-types reflect the actual development orders [9].

When examining the main lineage toward the terminal cell-type of granule cells, although all models capture the principle direction, DeepVelo can show a more consistent flow from the neurogenic intermediate progenitor cells (nIPC) to neuroblasts and finally to granule cells. DeepVelo particularly indicates that immature granule cells differentiate into mature granule cells in a manner more faithful to the true trajectory compared with the dynamical model (Fig. 2a - zoom-in panel).

The estimated velocities by DeepVelo show higher consistency in quantitative analysis. The consistency score is computed as follows - we first compute the average cosine similarity of the velocity vector of each cell to its neighbors, which is defined as the overall consistency. A similar neighbor-wise consistency was also proposed in scVelo [2]. However, the overall consistency could be biased toward over-smoothed estimations, which do not account for branching lineages. Therefore, we propose the cluster/cell-type-wise consistency as a complement to the overall score, which computes the average cosine similarity of each cell’s velocity to all velocity vectors of the same cell-type (“Overall and cell-type-wise consistency evaluation”). For both metrics, DeepVelo shows significant improvements over the scVelo dynamical method with significantly higher average consistency scores (Mann-Whitney *U* two-sided test $p < 1.0\times 10^{-300}$, $n=2930$ for both groups, Fig. 2b, c).

**Fig. 3:**
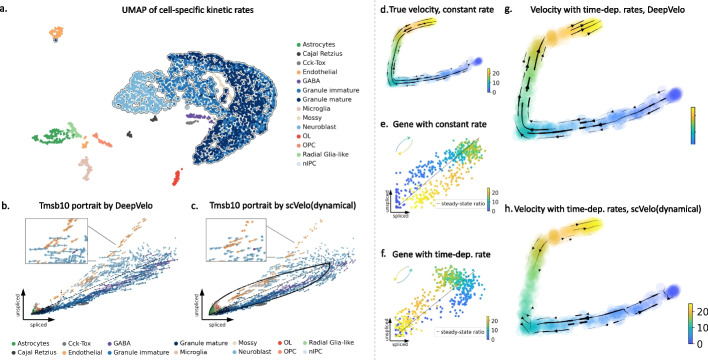
Velocity estimation for branching and time-dependent kinetic rates. **a** The UMAP projection of the estimated kinetic rates of 2930 cells in the dentate gyrus developmental data. Cells of the same cell-types are clustered together by kinetic rates. Furthermore, cells from the same lineage (e.g., the outlined Granule lineage) are positioned closely. In general, the similarity of learned kinetic rates reflects the biological similarity of cells, although the DeepVelo model is unaware of cell-type labels. **b** Projection of estimated velocity (arrows) onto the spliced/unspliced phase portrait of *Tmsb10* by DeepVelo. The endothelial cells undergo a separate trajectory on the phase portrait, aside from the main trajectory containing neuroblast cells, granule immature, and granule mature cells. DeepVelo successfully captures both trajectories. In the zoomed view, cells within the same region comprising of different cell-types are correctly predicted to have distinct velocity directions. **c** Phase portrait of *Tmsb10* with RNA velocity predicted by the scVelo dynamical model. Only the main trajectory of granule lineage is captured, but the endothelial cells are predicted with incorrect directions. **d**–**h** A simulation of time-dependent degradation rates. The cell color indicates its pseudotime in simulation. **d** Reference velocity with constant kinetic rates. **e**, **f** Constant and time-dependent degradation rates as shown on phase portraits. The gene with the time-dependent rate (**f**) undergoes a reversed trajectory. **g**, **h** Estimated velocities by DeepVelo and scVelo, respectively, for the simulated 500 cells with time-dependent degradation rates. DeepVelo correctly recovers the directions from regions of earlier time to later ones

Examined at the individual gene level, DeepVelo shows biologically meaningful velocity patterns. For example, *Tmsb10* is one of the major regulators to the inferred dynamics of granule lineage, and it plays an important role in the development of hippocampal CA1 region [18]. In Fig. 2f, velocities derived from the DeepVelo are consistent across velocities of neighboring cells. The region of cells showing high velocities of *Tmsb10* aligns well with the region of high *Tmsb10* expression. The same alignment is also observed in the example of another regulatory gene, *Ppp3ca* (Fig. 2g). In further analysis (Fig. 3a), we also observed that DeepVelo clearly disentangles the velocity vectors between the granule (blue) and endothelial lineages (orange), whereas, in the steady-state and dynamical models, both lineages have intertwined velocities. We discuss this advantage of cell-type-specific prediction in the next section.

Furthermore, this result demonstrates DeepVelo’s applicability to datasets that have multiple time points, and as such, may also contain batch effects. We further demonstrate DeepVelo’s applicability in these scenarios in subsequent Results sections involving multi-batch/temporal hindbrain and mesenchymal/chondrocyte organogenesis developmental datasets.

### DeepVelo’s cell-specific kinetic rates enable accurate quantification of time-dependent and multifaceted gene dynamics

Due to the cell-specific estimation of ($\alpha _{i,g}, \beta _{i,g}, \gamma _{i,g}$ in Eq. 1), DeepVelo for the first time provides a profile of individual kinetic rates for each cell. This enables new approaches for cell-specific trajectory analysis, visualization, and characterization. We show the UMAP projection of all cell-specific kinetic rates of 2930 cells (Fig. 3a). Although DeepVelo is unaware of the cell-types during training, the learned kinetic rates are naturally clustered into groups aligned with cell-types. Furthermore, clusters of cells from the same lineage (e.g., the outlined granule lineage) are positioned closely compared to other cells. Overall, the similarity of learned kinetic rates reflects the biological similarity of cells at both the cell-type and lineage levels. This indicates that DeepVelo can estimate kinetics that reflect the dynamics of individual cells as opposed to the entire dataset.

Velocity-associated kinetic rates across cells may vary for genes undergoing dynamic regulation involving multiple processes. For example, Battich et al. [19] observed varying kinetic rates in the differentiation of intestinal stem cells. These varying kinetics are often misinterpreted in existing velocity methods [20]. This stems from the fact that the kinetic rates in previous methods are modeled as constant cell-agnostic coefficients in first-order equations (Eq. 2), which lack the ability to model multifaceted dynamical variation. In contrast, DeepVelo estimates transcriptional dynamics for different cell-types and cell states by introducing cell-specific kinetic rates, leading to better velocity estimation in time-dependent and complex multi-lineage systems. Here, we show this improvement using two challenging scenarios:

(1) Estimating velocity for genes that are separately regulated in two lineages. We used the previously analyzed dentate gyrus cell population and determined genes with multifaceted kinetics [9]. *Tmsb10* shows multiple kinetic regimes and undergoes multiple trajectories. We plot the spliced and unspliced reads across all cells in this dataset, in other words, the phase portrait of *Tmsb10* (Fig. 2d). The cells in the granule lineage (including neuroblast, granule immature, and granule mature cell-types) form a cyclic trajectory. Meanwhile, the endothelial cells are not a part of the granule lineage and undergo a separate trajectory. These two regimes are likely regulated by different kinetic rates.

DeepVelo correctly predicted the RNA velocity patterns for both regimes (Fig. 3b). For the granule lineage, DeepVelo captures the direction of velocity from neuroblast cells to granule immature cells and then to granule mature cells. For the endothelial cells, the predicted velocity direction correctly points to the position of the same cell-type with amplified spliced reads. We also found that DeepVelo learns to assign similar velocity directions for cells of the same type. In contrast to DeepVelo, scVelo forces the RNA velocity to follow the cyclic trajectory assumed by the model (Fig. 3c). As a result, although scVelo successfully captures the trajectory for the granule lineage, it incorrectly points the velocity estimates of endothelial cells to the position of neuroblasts (Fig. 3c - Zoom-in panel).

Additionally, DeepVelo is capable of predicting distinct velocity directions for cells within the same region (Fig. 3b). The cells in the zoomed view, including both the endothelial and neuroblast cells, employ similar RNA dynamics (through the levels of spliced and unspliced reads) of *Tmsb10*. However, the distinct directions for each cell-type are correctly predicted by DeepVelo. This is due to the ability of DeepVelo to estimate distinct sets of kinetic rates across cell-types, as shown in Fig. 3a. In contrast, scVelo uses constant kinetic rates per gene and predicts a uniform direction for the same region of cells. Overall, a cell-specific model such as DeepVelo broadens the application of RNA velocity for genes with multifaceted kinetics, such as *Tmsb10* in the dentate gyrus developmental data.

(2) Estimating velocity for genes with time-dependent kinetic rates. We simulated a population of 500 cells and 30 genes using the simulator provided by the scVelo package [2]. We first determined the reference velocity in the setting of constant kinetic rates across cells (Fig. 3d, e). From here, the degradation rate, *gamma*, of 3 out of 30 genes was set to increase over time. As a result, the genes underwent a reversed trajectory as shown in the respective phase portrait (Fig. 3f). This simulation procedure of reversed dynamics was originally proposed in Bergen et al. [20], and it sets up a challenging scenario for the estimation of RNA velocity. The resulting velocity plots of DeepVelo and the dynamical model of scVelo are shown in Fig. 3g, h, and scVelo struggles to predict velocities from early to later time points while DeepVelo is able to recover the correct velocity directions from regions of earlier to later pseudotime. This advantage is because DeepVelo learns to find potential future cell states by integrating across all genes (“Continuity assumption and learning objectives”); thus, it is more robust to the time-reversed directions of a portion of genes in the dataset.

### DeepVelo infers functionally relevant lineage-specific genes and processes in hindbrain development

**Fig. 4:**
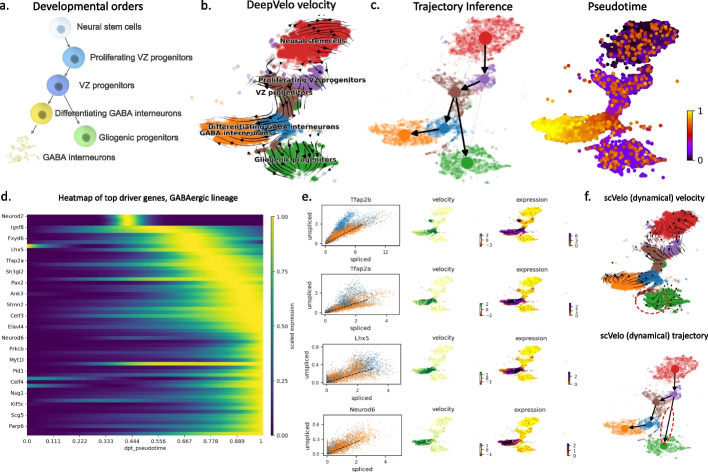
Velocity, trajectory, and driver gene estimation of developing mouse hindbrain cells. **a** The putative developmental order for six cell-types in early mouse hindbrain development. **b** The velocity projected onto the t-distributed stochastic neighbor embedding (tsne) plot of gene expression. DeepVelo’s RNA velocity reveals the temporal order in the developing mouse hindbrain, including cells from early progenitors, GABAergic, and gliogenic lineages. **c** Velocity-based PAGA trajectory inference using DeepVelo’s velocity estimates. The predicted trajectory correctly reflects the developmental relations shown in **a**. **d** The top 60 driver genes with highest correlation to the GABAergic lineage computed using DeepVelo’s velocity estimation. The horizontal coordinates represent the pseudotime estimates. **e** Gene phase portrait, velocity, and gene expression plots of selected driver genes. Known functional genes in the GABAergic lineage - *Tfap2b*, *Tfap2a*, *Lhx5*, and *Neurod6 *- are computed among the top driver genes. **f** The velocity plot and trjectory inference using the scVelo dynamical model

To test velocity methods in a complex setting with multiple lineages, we applied methods to a temporal mouse hindbrain development dataset [11] (Fig. 4a, Additional file 1: Fig. S4). Specifically, we filtered the data corresponding to the junction and differentiation between the GABAergic and gliogenic lineages (“Preprocessing the scRNA-seq data for DeepVelo”). In a multi-faceted system such as this, which is typical of developmental scRNA-seq datasets, considering cell-agnostic kinetic rates is haphazard because of the different RNA velocity dynamics among lineages. DeepVelo’s ability to learn cell-specific kinetic rates alleviates this assumption and accounts for the multi-faceted differentiation of the GABAergic and gliogenic lineages and their respective cell-types. The result of DeepVelo (Fig. 4b) shows the RNA velocity over the developmental process from Neural stem cells to the differentiating GABA interneurons and gliogenic progenitors. We performed trajectory inference using directional PAGA [21] over the velocity graph of DeepVelo. We found that DeepVelo was able to recapitulate ground-truth differentiation patterns - specifically the branching between VZ progenitors and differentiation GABA interneurons and gliogenic progenitors (Fig. 4c). The cluster of neural stem cells is well recognized as the origin cell-type with outward velocity arrows and a low pseudotime index, while the GABA interneurons are confirmed as a terminal cell-type with incoming velocity arrows and a high pseudotime index. In comparison, the scVelo dynamical model predicts partially inverse velocity directions for the gliogenic progenitors, leading to incorrect relations in the inferred trajectory (Fig. 4f, highlighted regions).

Using the velocity vector for each cell, we built a connectivity graph (“Computing cell-to-cell connectivity graph”) of the scRNA-seq data. CellRank [22] is a recent visualization and analysis toolbox for scRNA-seq data that utilizes the connectivity graph to predict cell’s fate mapping, which corresponds to the probability of the cell differentiating to a terminal state in the lineage(s). After determining cell fate, gene importance for differentiation can be calculated based on the correlation of gene expression with transition and differentiation probabilities towards all terminal states. The genes that display dynamical behavior across a lineage are termed putative “driver genes,” as these are the genes most likely to be involved in regulating the differentiation process itself. CellRank has been reported to work well with other velocity methods, such as scVelo, to infer lineage-specific drivers. We incorporated this toolbox with the predicted velocity connectivity graph from DeepVelo and determined driver genes in the variable gene subset of the data for both the GABAergic and gliogenic lineages.

Within the top 100 driver genes across both lineages of interest, we observed groups of genes showing particular abundance in specific cell-types in a temporal manner (Fig. 4d). For example, *Tfap2a*, *Tfap2b*, and *Lhx5*, which are two known differentiation genes involved in the specification of GABAergic interneurons during hindbrain development, are listed in the top 100 driver genes from DeepVelo for the GABAergic lineage (Fig. 4e) [23, 24]. Similar results were found for the gliogenic lineage from DeepVelo, with detection of known glial cell differentiation regulators in *Hes1* and *Sox9* (Additional file 2: Table S1) [25, 26]. DeepVelo also picked up hits that were novel and not detected by scVelo, such as *Neurod6* in the GABAergic developmental lineage (Fig. 4e). Although the role of *Neurod6* in the differentiating GABAergic interneurons and their development is unclear, previous literature has indicated the gene’s involvement in regulating the specification of inhibitory GABAergic interneuron subpopulations in the hindbrain and spinal cord [27]. This indicates a testable link and hypothesis for the differentiation of these cells in the junction within the GABAergic and gliogenic lineages, highlighting the ability of DeepVelo to guide searches of functional genes in scRNA-seq data and potential drivers of the differentiation process.

**Fig. 5:**
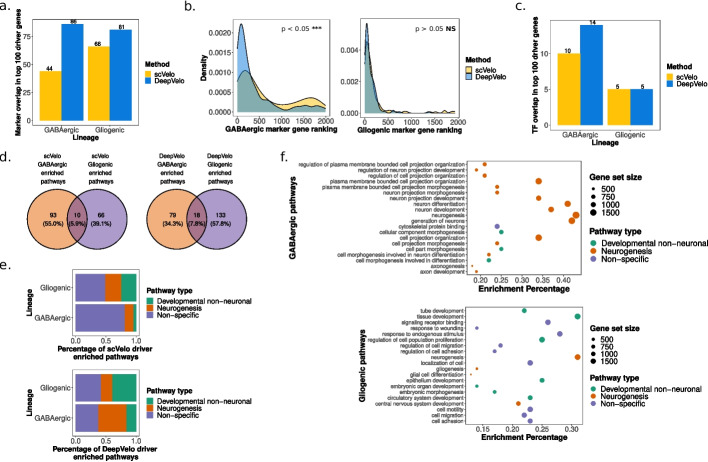
Functional enrichment of DeepVelo predicted driver genes. **a** Overlap of the top 100 driver genes from scVelo and DeepVelo for GABAergic and gliogenic lineages with annotated lineage marker genes. **b** Ranking density of marker-overlapping driver genes (across all 2000 tested genes) for scVelo and DeepVelo, separated by the GABAergic and gliogenic lineages, respectively. **c** Overlap of top 100 driver genes from DeepVelo and scVelo for both lineages with annotated transcription factors. **d** Pathway enrichment analysis results for the top 100 scVelo and DeepVelo driver genes, respectively, in the GABAergic and gliogenic lineages. **e** Functional signal in the enriched pathways for scVelo and DeepVelo, based on the presence of pathways involved directly in neurogenesis (“Neurogenesis”), not specific to neurogenesis but involved in development (“Developmental non-neuronal”), and not specific to either development or neurogenesis (“Non-specific”). **f** The top 20 DeepVelo pathway enrichment analysis results, based on FDR corrected *p*-values, for the GABAergic and gliogenic lineages, respectively

To compare the results of driver analysis when employing CellRank with different velocity outputs, we determined driver genes for the gliogenic and GABAergic lineages using both scVelo and DeepVelo (“Driver gene estimation and comparison”). As the complete set of genes driving differentiation in the complex hindbrain developmental system is unknown, we sought to infer the relevance of inferred driver genes in two ways: (1) by considering their overlap with predicted marker genes from the original analysis, as these genes are characteristic of cell-type identity and should be correlated with lineage specification, and (2) by considering their overlap with transcription factors (TFs), as TFs are the main elements responsible for differentiation and establishing transcriptional and cellular identity. We analyzed and compared the top 100 driver genes for both the GABAergic and gliogenic lineages predicted by the scVelo and DeepVelo methods (Additional file 2: Table S1). DeepVelo predicted driver genes that overlapped with more of the original markers from Vladoiu et al. [11], for both the GABAergic and gliogenic lineages (Fig. 5a) (Additional file 3: Table S2). Furthermore, to determine the signal across all driver genes, not limiting to the top 100, we determined the rankings of known marker genes in the GABAergic and gliogenic lineages across all tested driver genes. These rankings were determined based on the correlation scores, which indicate the relative importance of driver genes to a specific lineage. In this case, DeepVelo had higher rankings compared to scVelo for known GABAergic marker genes in the driver analysis (Mann-Whitney *U* two-sided test $p = 1.376\times 10^{-07}$, $n=245$ for both groups), while the ranking differences in the gliogenic lineage were non-significant ($p > 0.05$, $n=131$ for both groups) (Fig. 5b). When examining the transcription factor overlap in the top 100 driver genes, DeepVelo had more hits than scVelo for the GABAergic lineage and an equal number of hits for the gliogenic lineage (Fig. 5c).

For further examination of the results of driver analysis, we took the top 100 driver genes for the GABAergic and gliogenic lineages from DeepVelo and sought to determine their functional signal as gene-sets through pathway enrichment analysis (“Pathway enrichment analysis”). Overall, 97 and 151 pathways were found to be significantly enriched for the GABAergic and gliogenic lineages, respectively, for DeepVelo (Fig. 5d) (Additional file 4: Table S3). These pathways were analyzed for the presence of neurogenesis and developmental results, for which we did see a functional enrichment in both lineages (Fig. 5e). More specifically, the top 20 pathways for each lineage, ranked in terms of FDR-corrected *p* values, revealed enrichment of pathways relevant to neuronal differentiation processes (Fig. 5f). In the GABAergic lineage, enriched pathways included the following: *regulation of neuron projection development*, *neuron differentiation*, and *neurogenesis* (Fig. 5f). The results from the gliogenic lineage had even more relevant terms, namely *gliogenesis* and *glial cell differentiation* (Fig. 5f). When comparing these results with pathway analysis performed on the scVelo top 100 driver genes, we observed a much lower percentage of functional enrichment for neurogenesis and developmental pathways compared to DeepVelo for the GABAergic lineage (Fisher’s exact two-sided test $p = 1.407\times 10^{-09}$, $n = 103$ for scVelo and $n = 97$ for DeepVelo) (Fig. 5e), while the difference between the gliogenic results was non-significant ($p > 0.05$, $n = 76$ for scVelo and $n=151$ for DeepVelo). These functional pathway enrichment results highlight the relevance of the driver genes predicted by the DeepVelo method and increased functional relevance compared to those predicted by scVelo.

### DeepVelo outperforms current techniques in highly complex multi-furcating developmental data

**Fig. 6:**
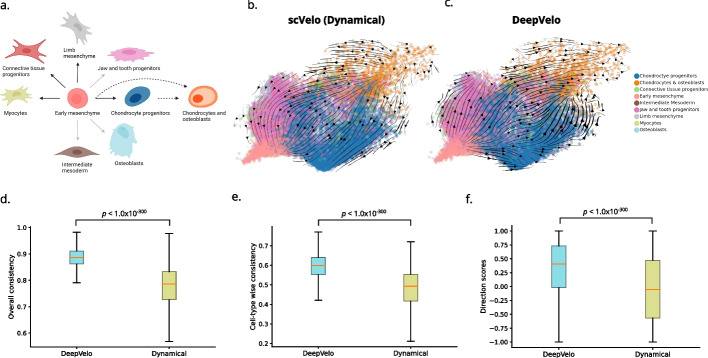
RNA velocity analysis of mouse mesenchymal and chondrocyte development. **a** For the mesenchymal/chondrocyte lineage, the differentiation trajectory as indicated by Cao et al. [12]. Solid black arrows indicate differentiation confirmed by the original paper, solid grey arrows indicate assumed differentiation paths, and dashed arrows indicate differentiation of a subset of the cells. **b**, **c** The RNA velocity estimates from the dynamical scVelo model (**b**) and the DeepVelo model (**c**) for the mesenchymal/chondrocyte lineage. **d**, **e** RNA velocity consistency values for overall consistency (**d**) and cell-type wise consistency (**e**) for the DeepVelo and dynamical scVelo model for the chondrocyte/mesenchymal lineage. **f** The direction scores for each individual cell in the chondrocyte/mesenchymal lineage for the DeepVelo and dynamic scVelo methods

As DeepVelo demonstrated strong performance and the ability to identify relevant driver genes in the hindbrain developmental data with a bifurcating lineage, a natural extension is to test in scenarios with multiple ($n>2$) furcations. To test DeepVelo’s ability to perform in these scenarios, we ran RNA velocity on the mesenchymal/chondrocyte lineage from the mouse organogenesis cell atlas (MOCA) dataset [12] (Fig. 6a). Within this lineage, early mesenchymal cells divide into several distinct cell-types, including myocytes, connective tissue progenitors, limb mesenchyme, jaw and tooth progenitors, chondrocyte progenitors, osteoblasts, and intermediate mesoderm (Fig. 6a).

DeepVelo was able to correctly predict both the correct direction and multi-furcating/branching differentiation paths (Fig. 6c). In particular, DeepVelo’s RNA velocity estimate indicated that the early mesenchymal cells are the progenitor state in this lineage, and they differentiate into both distinct and highly similar cell-types, including chondrocyte progenitors, jaw and tooth progenitors, and connective tissue progenitors (Fig. 6c). The dynamical scVelo model on the other hand did not predict the correct differentiation path, as it predicted the terminal chondrocyte and osteoblasts cell-type to be the progenitor state (Fig. 6c). This result is clearly indicated in the comparison of the direction scores based on the RNA velocity estimates (Fig. 6f) and their concordance with the differentiation lineages defined by Cao et al. (Fig. 6a). Furthermore, DeepVelo achieved higher overall and cell-type wise consistency scores (Fig. 6d, e).

As the mesenchymal/chondrocyte differentiation trajectory is multi-furcating, the genes involved in these processes are undergoing complex transcriptional dynamics that may not be captured by RNA velocity methods that do not consider both gene and cell-specificity for the calculation of transcriptional kinetic rates. DeepVelo’s strong performance on this dataset shows its applicability in scenarios where the differentiation dynamics are complex and the utility of cell-specific models for calculating RNA velocity.

### Analysis of pilocytic astrocytoma samples by DeepVelo reveals tumor subpopulations of varying immunogenicity

Pilocytic astrocytomas (PAs) are a class of low-grade gliomas that resemble astrocytic cells which typically localize in the cerebellum and are the most frequent class of brain tumor in patients within the ages of 0-19 years [28]. Although they are relatively benign tumors with good prognosis after surgical resection, they do have the potential to metastasize via the leptomeningeal route. Furthermore, in cases where complete surgical resection is not possible, chemotherapy and radiotherapy may be necessary, and these treatments can have adverse effects on the developing brains of the patients [28].

The most common genetic alteration within PAs is a gene fusion between KIAA1549 and BRAF [28]. This leads to a characteristic pathway alteration found in most PA samples, which affects the mitogen-activating protein kinase (MAPK) pathway [28]. Reitman et al. [29] further characterized the dysregulated gene programs using scRNA-seq data and discovered that PA tumor cells and oligodendrocyte precursor cells (OPCs) share gene expression signatures and that only a subset of PA cells express the MAPK program whereas the other cells express an astrocytic program that is indicative of more differentiated cells. Vladoiu et al. [11] leveraged both bulk and single-cell RNA sequencing data of normal hindbrain developmental data in mice and PA tumors from human patients to correlate normal developmental signatures with PA tumor markers and discovered that PA samples share signatures with normal gliogenic progenitor cells and the oligodendrocyte precursor cell lineage.

**Fig. 7:**
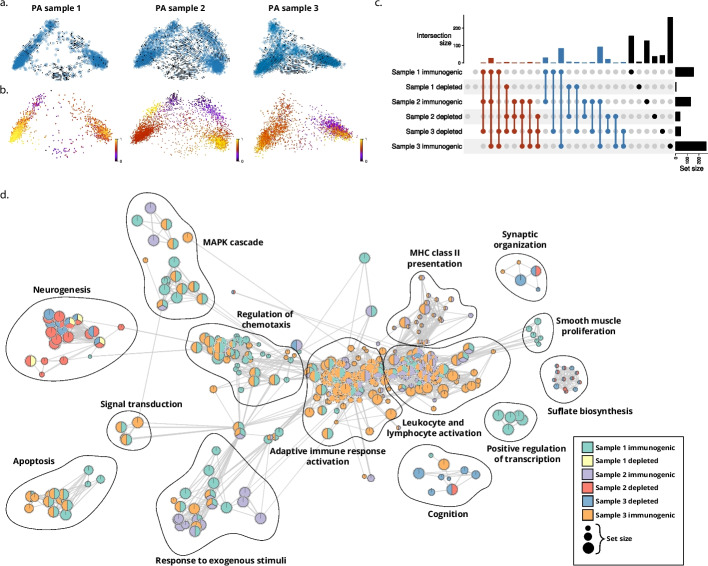
Analysis of branching dynamics in pilocytic astrocytoma (PA) tumor cells. **a** The DeepVelo RNA velocity results for tumor cells from the three PA samples. **b** The split-branch pseudotime results for the PA samples, indicating the early and late-stage tumor cells. **c** Upset plot showing set overlap of functionally enriched pathways, obtained from top driver genes, for branches within and across all samples. Branches are deemed “immunogenic” if they show enrichment for immune response related pathways and “depleted” otherwise. **d** Enrichment map of pathway enrichment analysis results for each of the two branches in the three PA samples. Each node is a pathway, colored by whether or not it was found to be statistically significantly enriched in the top 100 driver genes for each branch and sample. The size of the node indicates the size of the gene set/pathway

We sought to determine if RNA velocity analysis using DeepVelo can potentially discover novel biological signals in pilocytic astrocytoma. We analyzed three PA tumor samples from different patients with similar clinical and genomic characteristics (Additional file 6: Table S5) and performed RNA velocity analysis using DeepVelo exclusively on the PA tumor cells after filtering out the microenvironment and immune cell populations (“Preprocessing the scRNA-seq data for DeepVelo”). Within the PCA projection of the data, we observed two branches of tumor cells in each of the three samples, and the DeepVelo RNA velocity estimates reflected distinct branching dynamics in these samples (Fig. 7a.). We examined these distinct branches using both the discovered pathways from Reitman et al. [29] and normal human cerebellar development signatures from Aldinger et al. [30], but neither explained the variation that we observed (Additional file 1: Figs. S11-S16).

Given these distinct dynamics that are not correlated with previously observed variation in PAs, we performed pseudotime estimation individually for each branch (“Preprocessing the scRNA-seq data for DeepVelo”) for subsequent driver-gene analysis (Fig. 7b). After determining the driver genes for each branch, pathway enrichment analysis was performed on the top driver genes to determine any functional signatures that can differentiate the two branches in each sample (“Pathway enrichment analysis”). The results revealed that each branch has distinct functional signatures and that branches with a higher number of enriched pathways share functional signature across samples (Fig. 7c). Branches that had a strong functional enrichment signature were enriched for pathways indicating response to immune cells, such as *antigen processing and presentation*, *Cytokine Signaling in Immune system*, and *positive regulation of immune system process* (Additional file 7: Table S6). As we observed many of these pathways to be enriched, we broadly classified them into general programs such as “Adaptive immune response activation” (Fig. 7d). The other branches in each sample exhibited much less functional enrichment in terms of the number of significant pathways and were typically enriched in neurogenesis, synaptic organization, and biosynthesis pathways (Fig. 7d, Additional file 7: Table S6). As such, we deemed the immune signature enriched branches as “immunogenic” and the other branches as “depleted,” as the major differentiating signal observed in these two tumor cell branches was enrichment for immune response pathways (Fig. 7c, d). Furthermore, we also observed that MAPK pathways were only activated in the immunogenic populations (Fig. 7d), indicating that although the Reitman et al. marker genes did not show variation for the MAPK pathway across branches, there may still be some concordance between this program and the observed variation in immunogenicity. The discrepancy between the Reitman et al. marker scores and the pathway analysis results is likely because the Reitman et al. markers were curated for a PA-specific MAPK program [29], whereas the Gene Ontology (GO) and REACTOME pathways contain all MAPK associated factors. Furthermore, the driver gene analysis infers dynamic programs across pseudotime, whereas a marker-gene-based module score does not.

To the best of our knowledge, intra-tumor variation in immunogenicity and antigen presentation has not been previously reported in PAs. The role of the immune system in low grade gliomas such as PAs has not been studied extensively. Some studies [31, 32] have reported dysregulation of immune-related programs and varying levels of infiltration of effector immune cells, but the contribution of these factors to disease severity, presentation, and outcome have not been analyzed. Our analysis using DeepVelo indicates that there may be tumor compartments with distinct immunogenicity profiles which potentially arise from a common progenitor state. A variety of factors can contribute to low immunogenicity in tumor cells, such as restriction of antigen presentation, upregulation of immunoinhibitory pathways, and low levels of leukocyte infiltration [33]. The latter was not observed in the PA samples, as all three had high levels of microglia and T-cells in the microenvironment (Additional file 8: Table S7). Therefore, it is likely that modulation of antigenicity or immunoinhibitory programs is driving the variation in tumor cell immunogenicity.

To summarize, we discovered variation in PA tumor cells indicating significantly different levels of immune system activation, and this heterogeneity appears consistently in all three PA samples. Differing levels of immune system susceptibility of tumor cells has major implications in both prognosis and treatment of tumors, particularly those that require immunotherapy [34]. Although PAs are typically benign, the driving factors behind aggressive presentations are not well understood [28], and our findings warrant further analysis of tumor cell immunogenicity and their contribution to disease severity and prognosis. In higher grade gliomas, such as glioblastoma, variation in immunogenicity may be an even more important factor, and also warrants application and testing. Our findings demonstrate DeepVelo’s ability to pick up nuanced signal and generate novel biological hypotheses, particularly in cases where multifaceted dynamics are present. Furthermore, the findings were reproduced in three independent samples, indicating the robustness of DeepVelo’s results to technical variation and ability to recapitulate common biological signal across samples and datasets.

### DeepVelo is computationally robust and efficient across multiple scRNA-seq datasets

To examine the robustness of the DeepVelo RNA velocity estimates across settings, we tested DeepVelo on five different scRNA-seq datasets. Apart from the previously analyzed datasets, DeepVelo also recovers accurate RNA velocity vectors and developmental relations on a large-scale hippocampus data from La Manno et al. [1] (Additional file 1: Fig. S6). On all tested datasets, DeepVelo achieves higher average scores and lower variance in terms of the overall consistency compared to the scVelo dynamical model and the scVelo stochastic model (Additional file 5: Table S4).

We further tested the influence of multiple training, objective, and preprocessing hyperparameters on the dentate gyrus neurogenesis data (Additional file 1: Note S1, Additional file 1: Figs. S19-S27). DeepVelo is robust to changes in these hyperparameters and consistently estimates the biologically accurate RNA velocity, indicated by the resulting consistency and direction score values (Additional file 1: Figs. S19-S27). Particularly important is the fact that DeepVelo is robust to the choice of number of neighbors to use for the future time point state calculation (Additional file 1: Fig. S21). This calculation is central to the continuity assumption in DeepVelo (“Continuity assumption and learning objectives”), and this result highlights the robustness of continuity to hyperparameters central to the assumption.

Lastly, we compared the computational runtime of DeepVelo with other velocity estimation methods. Using the same CPU (central processing unit) device, DeepVelo (cpu) achieved a 4 fold speedup with respect to the scVelo dynamical model. Using a more powerful GPU (graphical processing unit) for the deep learning backbone, DeepVelo (cpu+gpu) can be further accelerated 10–20 times across datasets. For example, DeepVelo completed the training and estimation for the 13501 cells of developmental hindbrain data in just 36 seconds (Additional file 1: Fig. S8).

## Discussion

DeepVelo offers a novel velocity estimation framework that goes beyond assumptions of constant RNA splicing and degradation rates and instead estimates these rates at a cell-specific level. By analyzing the performance of DeepVelo and existing velocity estimation techniques, we have demonstrated that DeepVelo’s cell-specific estimation through a novel deep learning method allows for the detection and specification of multiple lineages in calculating RNA velocity. Realistic single-cell RNA sequencing settings will likely have more than one lineage/trajectory in a given sample, and thus it is imperative to develop methods that can account for these multifaceted dynamical systems. DeepVelo’s ability to model these multifaceted dynamics was demonstrated through analysis of complex differentiation systems, such as the development of the dentate gyrus, pancreatic endocrinogenesis, and chondrocyte development. Lastly, we demonstrated that DeepVelo can be utilized to identify functionally relevant genes that are enriched along multi-furcating differentiation trajectories, in systems including hindbrain development and pilocytic astrocytoma. We envision that DeepVelo will be more readily applicable to these realistic developmental settings as compared to previous techniques.

DeepVelo internally predicts the first-order derivative of expression per gene based on the transcriptome-wide reads of all selected genes. The ability to learn the interaction/regulation between genes could be further explored, for example, by replacing the GCN model with recent transformer networks [35] which could explicitly model the interaction of internal gene representations. This could allow for more interpretable velocity and driver-gene estimates, by considering correlations of kinetics and expression patterns between genes and cells. Recent work shows promising research directions by extending the velocity of cellular dynamics from RNA to proteins [6], epigenomics [5], and multi-omics velocities [4]. DeepVelo could be naturally updated and well fitted into these settings by enriching input and output space with additional -omics information. Ultimately, the estimation of cell-specific kinetics across multiple steps in the central dogma may increase the signal-to-noise ratio [20] and further accurately capture information related to cellular development.

The continuity assumption is central to the DeepVelo model, and although this assumption allows DeepVelo to be more expressive and less constrained, it does have its limitations. One potential limitation is the applicability of DeepVelo to cell-types that may be less prevalent or difficult to characterize, such as rare cell-types. Finding the correct future time point neighbors for these cell-types may be challenging, thus hampering the applicability of the continuity assumption. However, we do show that the DeepVelo model is robust to the choice of neighbors in the future time point state calculation. Furthermore, DeepVelo works in a manner that is not biased by cell-type annotation, and as such, results will not be affected by incorrect annotation of rarer cell-types. Regardless, the challenge of modeling RNA velocity for rare cell-types is an important future research direction for RNA velocity methods, irrespective of their central assumptions, and future studies should emphasize the inclusion of data with less studied/rare cell-types.

Although DeepVelo demonstrates the efficacy of RNA velocity estimates on individual cell and gene levels, building a comprehensive theorem to verify the confidence of velocity estimation remains a major challenge. Empirical metrics, such as the consistency of velocity directions among neighboring cells have been used in this work and existing approaches [2, 7]. However, there is a lack of probabilistic tools to test the kinetics estimated by either previous methods or DeepVelo. We anticipate future work in the estimation of RNA velocity will address this gap and incorporate better approaches to uncertainty estimation in this field.

## Conclusions

RNA velocity techniques have allowed for insights into biological differentiation from single-cell RNA sequencing data that go beyond oversimplified trajectory inference models and instead infer dynamic processes that indicate the direction and magnitude of differentiation potential. Although many major limitations and assumptions for RNA velocity methods still exist, we anticipate that continued methodological development in this field will lead to better tools to study differentiation and development in a single-cell setting. DeepVelo overcomes limitations of previous techniques in a major aspect with regards to cell-specific model estimates and the continuity assumption. Our experiments on both normal developmental and tumor data demonstrate DeepVelo’s ability for more robust velocity estimation in multi-lineage systems, yielding better biological insights into real and complex biological use cases.

## Methods

### Preprocessing the scRNA-seq data for DeepVelo

The dentate gyrus neurogenesis [9], mouse hippocampus [1], and pancreatic endocrinogenesis [10] data are available at the NCBI Gene Expression Omnibus (GEO) repository. The accession numbers are GSE95753, GSE104323 and GSE132188. The mouse gastrulation [13] is available at https://github.com/MarioniLab/EmbryoTimecourse2018. In this work, we use the zipped data of these sequencing datasets provided by the scVelo package [2] (https://scVelo.org). The data is in h5py file format and contains spliced and unspliced gene readout.

Mouse hindbrain developmental data from [11] was used to test velocity techniques for estimation at a lineage junction. BAM files from GSE118068 were converted to fastq format using *bamtofastq* function from CellRanger. Fastq files were processed into loom files using kallisto reference-free alignment through the loompy pipeline [36]. This was done individually for each timepoint (E10, E12, E14, E16, E18, P0, P5, P7, P14), and processed loom files were concatenated. For the purposes of the analysis, the junction between the GABAergic and gliogenic lineages was utilized. The following cell-types were subset from time points E10, E12, E14, E16, E18, P0, P5, P7, and P14 - neural stem cells, proliferating VZ progenitors, VZ progenitors, differentiating GABA interneurons, gliogenic progenitors, and GABA interneurons. Estimates of spliced and unspliced counts from the kallisto quantification method were used for testing DeepVelo and scVelo.

The mouse organogenesis data from [12] was reprocessed using a pipeline from Alevin-Fry [37], using version 0.8.0 of the tool. The NCBI accession for the fastq files for this data is GSE119945 and is associated with the SRA archive PRJNA490754. The indicated pipeline is used to process all downloaded fastq files. We kept the processed “spliced,” “unspliced,” and “ambiguous” reads for RNA velocity analysis. Cells from the chondrocyte trajectory (subtrajectory name - chondrocyte trajectory) were used in the analysis. Cell-type annotations and UMAP coordinates for velocity projection were used from the Cao et al. study metadata. Prior to velocity inference using the reprocessed spliced/unspliced counts, cells from this trajectory were downsampled to 30000 cells for faster inference and easier interpretability of velocity portraits.

Three pilocytic astrocytoma (PA) tumor samples from the same study by Vladoiu et al. [11] were utilized for the PA RNA velocity analysis. The EGA accession for this data is EGAS00001003170. Using CellRanger version 7.0.1, BAM files were converted to fastq format using the *bamtofastq* function, and then the *count* function was utilized to obtain the quantified scRNA-seq count matrices - both with default parameters. The hg19 reference was used as it was the reference the original fastqs were mapped to. For running RNA velocity, conversion from BAM to loom format was done using the Velocyto package with default parameters [1].

The PA samples were filtered at the cell-level such that the number of non-zero genes per cell is > 200 and that the mitochondrial genome percentage is < 5% [38]. The data was also log-normalized using Seurat’s “LogNormalize” method with a “scale.factor” of 10000 [38]. A marker-gene list from Vladoiu et al. that was used to annotate clusters from the PA scRNA-seq samples in the original paper was used [11]. These clusters were annotated as “tumor cells,” “microglia/monocytes,” or “*t*-cells,” with different cluster numbers. Using the top 25 markers from each of the clusters’ DE lists, Seurat’s “AddModuleScores” function [38] was used to rank the cells from the PA samples for the respective clusters, and the top scoring module for each cell in each sample was used to annotate the cell. Afterwards, the granular cluster numbers were omitted, and annotations were collapsed to “tumor cells,” “microglia/monocytes,” or “*t*-cells.” Only the tumor cells were selected from each sample for subsequent analysis.

Module scores for the Aldinger et al. [30] developmental cell-types and Reitman et al. [29] PA programs (MAPK, AC, OC) in PA tumor cells were calculated. To score the programs from Reitman et al., the modules marker genes were used with the Seurat “AddModuleScore” function [38] and the default parameters for Seuratv4. As the marker list for the Aldinger et al. cell-types was based on differential expression (DE), subsetting was done for the top 25 markers (based on DE) for each cell-type. Afterwards, the same steps were followed as for the Reitman et al. analysis and the Seurat “AddModuleScore” function [38] was used to determine signatures of each cell-type in the tumor data.

Processing of unspliced and spliced counts in differing formats across datasets was done via three steps and using the scVelo package [2]. First, the spliced and unspliced gene matrices were normalized across genes. In more detail, preprocessing includes expression matrix normalization and nearest-neighbor-based smoothening. We used the scv.pp.filter_and_normalize function from scVelo for these steps with default parameters [2]. We selected the top 2000 or 3000 highly variable genes based on the spliced reads. The principal components are computed afterward using logarithmized spliced counts, and then the expression reads are smoothened using the average of 30 nearest neighbors for each cell [2].

### Modeling individual transcriptional dynamics

Transcriptional dynamic depicts the process from generation to degradation of mRNA molecules. It captures unspliced premature mRNAs u(t) with transcription rate $\alpha$, splicing into mature mRNAs *s*(*t*) with rate $\beta$ and the degradation of spliced mRNA with rate $\gamma$. The simplified gene-specific dynamics with constant splicing and degradation rates are

2 $$\begin{aligned} {\frac{{du\left( t \right) }}{{dt}} = }{} & {} {\alpha \left( t \right) - \beta u\left( t \right) ,} \nonumber \\ {\frac{{ds\left( t \right) }}{{dt}} = }{} & {} {\beta u\left( t \right) - \gamma s\left( t \right) .} \end{aligned}$$

This equation is used in existing velocity estimation methods, and it omits the difference in kinetic rates ($\alpha , \beta , \gamma$) across cell-types. Instead, we propose a new deep learning method to capture individual cell kinetics.

First, we build a graph convolutional network model to predict cell-specific kinetic rates. In this work, we employ a nearest neighbor graph based on the gene expression of all cells $G = (\mathcal {V}, \mathcal {E})$. The vertex $\mathfrak {v}_i \in \mathcal {V}$ in the graph denotes the expression reads of a cell *i*, which include its spliced and unspliced gene expression $\mathfrak {v}_i = [s_i, u_i]$. A cell *i* is connected to cell *j* (i.e., $\mathcal {E}_{ij} = 1$) if cell *j* is one of the top 30 nearest neighbors based on the Euclidean distance of gene expression. We input this neighbor graph to the DeepVelo model. We chose the graph representation because it considers the vicinity of local cells’ based on gene expression. This has more expressive power than the expression of individual cells because of the sparse and noisy nature of single-cell RNA sequencing counts. Taking the neighborhood expression into account smoothens the velocity estimation.

A graph convolutional network (GCN) is a type of deep neural networks that learns node embeddings based on message passing along the graph edges [39]. Given a graph with nodes $\mathcal {V}$ and adjacency matrix *A*, a multi-layer neural network is constructed on the graph with the following layer-wise propagation rule:

3 $$\begin{aligned} H^{(l+1)} = \sigma \left(\tilde{D}^{-\frac{1}{2}}\tilde{A}\tilde{D}^{-\frac{1}{2}}H^{(l)}W^{(l)}\right), \end{aligned}$$

where $H^{(l)}$ denotes the node feature vectors at the *l*-th layer, $\tilde{A} = A + I_{N}$ is the adjacency matrix with self-connections, $\tilde{D}$ is the diagonal degree matrix such that $\tilde{D}_{ii} = \sum _j \tilde{A}_{ij}$, $W^{(l)}$ is the layer-specific trainable parameter matrix, and $\sigma$ is the RELU activation function.

In this work, the input feature $H^{(0)} \in \mathbb {R}^{N \times 2D}$ to the GCN is the cell by gene count matrix. Each row in *H* stands for the aforementioned vertex $\mathfrak {v}_i$. *H* contains the population of *N* cells, and the dimension 2*D* equals the number of selected spliced and unspliced genes combined, $D=2000$ by default. The adjacency matrix $A \in \mathbb {R}^{N \times N}$ depicts the aforementioned nearest neighbor graph, where the element at position *i*, *j* has value 1 if the cell *j* is one of the nearest neighbors of cell *i*, otherwise the value is 0. The GCN model consists of stacked graph convolution layers, i.e., Eq. 3. The output of the final layer $H^{L}$ is processed by a fully connected neural network, which then yields the estimated velocity parameters $\alpha \in \mathbb {R}^{N \times D}$, $\beta \in \mathbb {R}^{N \times D}$ and $\gamma \in \mathbb {R}^{N \times D}$ for all cells and genes.

Finally, the estimated velocity $\tilde{v}_i \in \mathbb {R}^{D}$ for each cell is computed as

4 $$\begin{aligned} \tilde{v}_i = \beta _i u_i - \gamma _i s_i, \end{aligned}$$

where $\beta _i$ and $\gamma _i$ are the *i*-th row in $\beta$ and $\gamma$, $u_i$ and $s_i$ are the unspliced and spliced reads of cell *i*.

DeepVelo also supports estimation of the derivative of unspliced RNA, namely $v^{uns}_i$, which is an estimation for the $\frac{du(t)}{dt}$ in Eq. 1.

$$\begin{aligned} \tilde{v}^{uns}_i = \alpha _i - \beta _i s_i. \end{aligned}$$

### Continuity assumption and learning objectives

In this section, we propose a learning framework for RNA velocity to optimize the velocity estimates in Eq. 4, and then introduce the specific training objective following this framework.

#### Extrapolating cell states along time based on the continuity assumption

RNA velocity is defined as the time derivative of spliced mRNA (Eq. 1). For a specific cell *i* out of the sequenced cell population $\Omega$, the velocity vector $v_i$ contains the derivative for all genes, as

5 $$\begin{aligned} v_i := \frac{ds_i}{dt} = \left[\frac{ds^{(1)}_i}{dt}, \frac{ds^{(2)}_i}{dt}, \dots \frac{ds^{(|D|)}_i}{dt}\right], \end{aligned}$$

where $s^{(g)}_i$ denotes the amount of spliced mRNA of one gene. $s_i$ is the spliced gene expression vector containing $\left[s^{(1)}_i, s^{(2)}_i, \dots , s^{(|D|)}_i\right]$.

We introduce the multivariate random variable $G_{i,\tau (i)}$ to represent the (spliced) gene expression that a cell *i* could have at its developmental time $\tau (i)$, where $\tau$ is an operator to obtain a cell’s current time in its developmental process. Thus, the scRNA-seq results could be viewed as an observation of $G_{i,\tau (i)}$ taking the value $s_i$. For simplicity, let us use $t=\tau (i)$ as the time of cell *i*. Similarly, we define the random vector $V_{i,t}$ as the possible velocities that cell *i* can take at time *t*, and $v_i$ is an observation of $V_{i,t}$. The relation between the expression and velocity random vectors is,

$$\begin{aligned} V_{i,t} = \frac{dG_{i,t}}{dt}. \end{aligned}$$

We can use the forward difference to approximate the derivative if the time interval is sufficiently small, as

6 $$\begin{aligned} V_{i,t} \approx \Delta G_{i,t} = G_{i,t+1} - G_{i,t}. \end{aligned}$$

Notably, it is impossible to directly observe the future stage $G_{i,t+1}$ for cell expression from scRNA-seq, because the sequencing protocol is destructive, and cells no longer exist after sequencing. Thus, the estimation of $G_{i,t+1}$ is required.

DeepVelo utilized the mRNA expression of neighboring cells to estimate $G_{i,t+1}$ and for this reason, we introduce the *continuity assumption*: we assume that the sequencing data captures a continuous spectrum of cells in consecutive differentiation/developmental stages. Particularly, there exists a $t+1$ neighborhood, $\mathcal {N}_{i,t+1}$, in the sequenced cell population, so that the gene expression of these cells within the neighborhood are similar enough to the potential expression of cell *i* at $t+1$. In other words, the expected expression of the $t+1$ neighbor cells have the same distribution as the expression of cell *i* at $t+1$. In comparison to the previous strict assumptions (i.e., the observation of steady states or the global constant kinetic rates) in existing approaches, the continuity is primarily satisfied in high-throughput scRNA-seq data of large cell populations. Formally, continuity can be expressed as

7 $$\begin{aligned} \forall i \in \Omega , \quad \exists{} & {} \mathcal {N}_{i,t+1} \subset \Omega , \quad s.t. \nonumber \\ G_{i,t+1}{} & {} = \sum \limits _{j \in \mathcal {N}_{i,t+1}} G_{j,\tau (j)}P(i \rightarrow j) \nonumber \\{} & {} = \mathbb {E}_{P(i \rightarrow j)}[G_{j,\tau (j)}], \end{aligned}$$

where $i \rightarrow j$ denotes that cell *i* develops at time $t+1$ into a cell that has the same gene expression vector as cell *j*, and $P(i \rightarrow j)$ is the probability of this event. The expectation of $G_{i,t+1}$ over all cells in the sequenced population $\Omega$ is

8 $$\begin{aligned}{} & {} \mathbb {E}_{i\in \Omega}\left[ G_{i,t+1}\right] = \mathbb {E}_{i\in \Omega }\left[ \mathbb {E}_{P(i \rightarrow j)}[G_{j,\tau (j)}]\right] \nonumber \\{} & {} \mathbb {E}_{i\in \Omega }\left[ G_{i,t+1} - \mathbb {E}_{P(i \rightarrow j)}[G_{j,\tau (j)}] \right] = 0, \end{aligned}$$

Taking in Eq. 6, we have

9 $$\begin{aligned} \mathbb {E}_{i\in \Omega }\left[ G_{i,t} + V_{i,t} - \mathbb {E}_{P(i \rightarrow j)}[G_{j,\tau (j)}] \right] = 0. \end{aligned}$$

The observed sequenced expression in a large cell population can be used to derive the Monte Carlo estimator of the outer expectation over cell *i*. Assuming each cell’s expression vector $s_i$ is independent,

10 $$\begin{aligned} \frac{1}{\Omega } \sum \limits _{i \in \Omega }\left[ s_i + v_i - \sum \limits _{j \in \mathcal {N}_{i,t+1}} s_j P \left( i \rightarrow j \right) \right] \approx 0. \end{aligned}$$

Because the $v_i$ and $\mathcal {N}_{i,t+1}$ are not directly observed, given a set of estimated $\tilde{v}_i$ and $\mathcal {\tilde{N}}_{i,t+1}$, we use the (gene-wise) squared difference as an objective to measure how close to zero the value in Eq. 10 is.

11 $$\begin{aligned} \mathcal {L} = \frac{1}{\Omega } \sum \limits _{i \in \Omega }\left[ s_i + \tilde{v}_i - \sum \limits _{j \in \mathcal {\tilde{N}}_{i,t+1}} s_j P \left( i \rightarrow j \right) \right] ^2. \end{aligned}$$

This equation provides a general objective for any RNA velocity methods that generate the estimation of $\tilde{v}_i$, $\mathcal {\tilde{N}}_{i,t+1}$ and $P(i \rightarrow j)$.

#### Training the DeepVelo model

We follow the Eq. 11 to develop the objective to optimize the parameters of the DeepVelo model. The objective computes the difference between the estimated velocity $\tilde{v}_i$ (Eq. 4) of DeepVelo and possible future cell states.

We first select $K_c$ number of nearest neighbor cells for each cell *i* by computing the pairwise distances of spliced gene expression. By default, we compute the Euclidean distance of the first 30 PCA dimensions of the spliced counts. These selected cells compose the neighborhood of cell *i*, i.e., $\mathcal {N}_i$. We estimate the $P(i \rightarrow j )$ using

12 $$\begin{aligned} P_{c+}(i \rightarrow j ) = \left\{ \begin{array}{ll} \frac{1}{Z} &{} \text { if } S_{cos}(s_j-s_i, \tilde{v}_i)>0 \text { and } j\in \mathcal {N}_i,\\ 0 &{} \text { otherwise}, \end{array}\right. \end{aligned}$$

where $S_{cos}$ denotes the cosine similarity and *Z* is a normalizing factor, i.e., *Z* equals to number of cells in $\mathcal {N}_i$ satisfying $S_{cos}(s_j-s_i, \tilde{v}_i)>0$. The intuition of $P_{c+}$ is that if the scRNA-seq data satisfies the continuity assumption and the time interval between *t* and $t+1$ is small enough, then the possible future cell state $j \in \mathcal {N}_{i,t+1}$ is also close to the cell state of current cell *i*. Therefore, given a sufficient large $K_c$, $\mathcal {N}_{i,t+1} \subset \mathcal {N}_i$. Further in Eq. 12, We use the cosine similarity between the estimated velocity $\tilde{v}_i$ and the expression difference $s_j - s_i$ to select the possible target cell *j* that aligns with the velocity direction.

Notably, the Eq. 6 is the forward difference operation. Similarly, we can also include the backward difference $V_{i,t}= G_{i,t} - G_{i,t-1}$ and project the cell *i* into $t-1$. We first compute the probability that cell is *i* differentiated from cell *j*, $P_{c-}(i \leftarrow j)$, as follows

13 $$\begin{aligned} P_{c-}(i \leftarrow j ) = \left\{ \begin{array}{ll} \frac{1}{Z} &{} \text { if } S_{cos}(s_j-s_i, -\tilde{v}_i)>0 \text { and } j\in \mathcal {N}_i,\\ 0 &{} \text { otherwise}. \end{array}\right. \end{aligned}$$

We then used this in the computation of $\mathcal {L}_-$ in Eq. 14. The sum of $\mathcal {L}_+ + \mathcal {L}_-$ is symmetric to either $\tilde{v}_i$ or $-\tilde{v}_i$, which creates a challenge to determine the correct velocity direction. To resolve this issue, we know from Eq. 1 that the velocity across cells should be positively correlated to the unspliced expression, $u_i$, and negatively correlated to the spliced expression, $s_i$. We discuss the properties and applicability of the Pearson correlation heuristics in Additional file 1: Note S4. We add the Pearson correlation in Eq. 14 term $\mathcal {L}_{Pearson}$ to promote determining the correct direction for the RNA velocity estimates. The aforementioned objective terms are as follows

14 $$\begin{aligned} \mathcal {L_+}{} & {} = \frac{1}{\Omega } \sum \limits _{i \in \Omega }\left[ s_i + \tilde{v}_i - \sum \limits _{j \in \mathcal {\tilde{N}}_{i}} s_j P_{c+} \left( i \rightarrow j \right) \right] ^2, \nonumber \\ \mathcal {L_-}{} & {} = \frac{1}{\Omega } \sum \limits _{i \in \Omega }\left[ s_i - \tilde{v}_i - \sum \limits _{j \in \mathcal {\tilde{N}}_{i}} s_j P_{c-} \left( i \leftarrow j \right) \right] ^2, \nonumber \\ \mathcal {L}_{Pearson}{} & {} = - \left( \lambda _u corr(\tilde{v}_i, u_i) + \lambda _s corr(\tilde{v}_i, - s_i)\right) , \end{aligned}$$

where *corr* denotes the Pearson correlation coefficient. We use the combination of the objective terms $\mathcal {L}_c = \mathcal {L_+} + \mathcal {L_-} + \mathcal {L}_{Pearson}$ to train the DeepVelo model. $\lambda _u, \lambda _s$ are constant factors to balance the scale of the objective terms. The model parameters are optimized to minimize $\mathcal {L}_c$.

Notably, for each gene, the optimization *integrates the information of other genes*, because the estimated target cell probability $P(i \rightarrow j)$ considers the full gene expression of cell *i* and *j*. From a per gene estimation perspective, it corrects the target cell probability when the unspliced/spliced counts of the current gene are noisy with respect to the true direction, but the majority of genes point to the correct target cell *j*. This integration of genes is a unique advantage of DeepVelo compared to existing methods, and it particularly contributes to the capability of cell-type-specific velocity prediction and time-dependent gene dynamics of DeepVelo (“DeepVelo’s cell-specific kinetic rates enable accurate quantification of time-dependent and multifaceted gene dynamics” section).

#### The continuity assumption relaxes previous constraints

The continuity assumption works in the situation when sufficient sequenced cells are present such that the future neighbors in Eq. 7 are guaranteed to exist. We prove in Additional file 1: Note S2 that the satisfaction of previous steady-state or constant-kinetics assumptions in such sufficient observed scenarios implies the satisfaction of the continuity assumption, and furthermore, in other scenarios where sequenced cells are insufficient, we proved that optimization using the continuity assumption will have smaller asymptotic error with respect to the number of sequenced cells. In other words, the continuity assumption relaxes the specific requirements on the gene dynamics in previous approaches and broadens scenarios for calculation of RNA velocity estimates.

### Implementation details

DeepVelo is implemented using Deep Graph Library (DGL) and Pytorch. The default model uses two hidden graph convolution layers of size 64. A dropout probability of 0.2 is used, with RELU activations between hidden layers. For each cell (individual data input into the model), the $\beta$ and $\gamma$ parameters are predicted, and the velocity is estimated as outlined in the “Modeling individual transcriptional dynamics” section. The Adam optimizer [40] is used with a learning rate of 0.001, no weight decay, and AMSGrad enabled. A learning rate decay scheduler is used that decays the learning rate by a gamma term of 0.97 after each training epoch. Full-size batch training (with batch size equal to the number of cells) and 100 training epochs are utilized by default. The values for the loss scaling ($\mathcal {L}_c = \mathcal {L_+} + \mathcal {L_-} + \mathcal {L}_{Pearson}$) are 1.0, 1.0, and 18.0 for each term, respectively. Through experiments on different datasets, we have found these default parameters to be sufficient in ensuring convergence and avoiding overfitting. Robustness to model and optimization hyperparameters is explored further in Additional file 1: Note S1.

### Continuity score as a confidence measure for velocity estimation

We use the difference between the final velocity estimates and the expected future cell states as an indicator of estimation error. After training and predicting on a given dataset, we compute the score per cell and gene as,

15 $$\begin{aligned} \epsilon ^{(con)}_{i,g} = \frac{1}{s_{i,g}} \cdot |s_{i,g} + \tilde{v}_{i,g} - \sum \limits _{j \in \mathcal {\tilde{N}}_{i,t+1}} s_{j,g} P \left( i \rightarrow j \right) |, \end{aligned}$$

Note this follows the inner term of Eq. 11, and we add the subscript *g* to indicate the gene it is computed on. Conceptually, this measures *how close the current estimates satisfy the continuity assumption*.

A cell-wise continuity score, CS-cell, is then computed as a measure of uncertainty for the average estimates of individual cells.

16 $$\begin{aligned} \texttt {CS-cell}_i = 1 - \frac{1}{M} \sum ^M_{g = 1} \texttt {tanh}\left( \epsilon ^{(con)}_{i,g} \right) . \end{aligned}$$

Similarly, a gene-wise continuity score, CS-gene, is introduced as the average continuity score in all cells.

17 $$\begin{aligned} \texttt {CS-gene}_g = 1 - \frac{1}{|\Omega |} \sum \limits _{i \in \Omega } \texttt {tanh}\left( \epsilon ^{(con)}_{i,g} \right) . \end{aligned}$$

We use the cell-wise and gene-wise continuity scores to indicate the confidence values for our velocity estimation. For example, we visualize the regions of cells where DeepVelo has more confidence (Additional file 1: Fig. S2), and we use continuity scores to filter out unfitted genes from downstream applications (Additional file 1: Note S4). Throughout the manuscript, we use the terms confidence score and continuity score interchangeably.

### Correlation score

After the training phase, we compute the Pearson correlation in Eq. 14 on the final velocity estimates to examine the actual correlation between the estimates and current gene expression. A higher correlation score indicates that it is more in line with our heuristic that velocity is correlated to the unspliced reads and anti-correlated to spliced reads.

### Overall and cell-type-wise consistency evaluation

The overall consistency score is the average cosine similarity of the velocity vectors to their neighbors. For each cell *i*,

$$\begin{aligned} C_{overall}(i) = \frac{1}{|\mathcal {N}^{(s)}_i|} \sum \limits _{j \in \mathcal {N}^{(s)}_i} S_{cos}(\tilde{v}_i, \tilde{v}_j), \end{aligned}$$

where $\mathcal {N}^{(s)}_i$ is the 30-nearest-neighbor cells with similar spliced gene expression, computed in the preprocessing step (“Preprocessing the scRNA-seq data for DeepVelo”). $S_{cos}$ denotes the cosine similarity operation. $v_i, v_j$ are the estimated velocities from Eq. 4.

The cell-type-wise consistency computes the similarities for each cell-type instead. For each cell *i* and associated cell-type $\mathcal {T}(i)$,

$$\begin{aligned} C_{cell-type} = \frac{1}{|\mathcal {T}(i)|} \sum \limits _{j \in \mathcal {T}(i)} S_{cos}(\tilde{v}_i, \tilde{v}_j), \end{aligned}$$

where $|\mathcal {T}(i)|$ denotes the number of cells belonging to the cell-type.

### Direction evaluation with annotated inter-cell-type relations

To evaluate velocity estimates based on known biological relations between cell-types, we measure the alignment between estimated direction and annotated cell-type relations. This direction score is inspired by the CBDir metric in [7]. Here, we introduce its original workflow and then our modification.

The metric requires annotation of directional pairs of cell-types, e.g., (A $\rightarrow$ B), and it is computed within the cross-boundary cells between A and B. For each given pair of cell-type annotations (A, B), the boundary cells are selected as:

18 $$\begin{aligned} \mathcal {N}_{A \rightarrow B} = \{i \in \mathcal {T}_A | \mathcal {N}(i) \cap \mathcal {T}_B \ne \emptyset \}, \end{aligned}$$

where $\mathcal {T}_A, \mathcal {T}_B$ denote the cells belonging to type A and type B, respectively. $\mathcal {N}(i)$ is the neighboring cells of cell *i*. We use the same neighbors from the preprocessing steps (“Preprocessing the scRNA-seq data for DeepVelo”). Then, the direction score for each boundary cell is

19 $$\begin{aligned} DS(i) = \frac{1}{|\mathcal {N}(i) \cap \mathcal {T}_B|} \sum \limits _{j \in \mathcal {N}(i) \cap \mathcal {T}_B} \frac{\tilde{v}_j \cdot (s_j - s_i)}{|\tilde{v}_j||s_j - s_i|}, \quad i \in \mathcal {N}_{A \rightarrow B}. \end{aligned}$$

Conceptually, the term $\frac{s_j - s_i}{|s_j - s_i|}$ captures the direction from the computed cell *i* along the annotated developing direction A $\rightarrow$ B. This direction score considers the average cosine similarities among all neighbors. A higher direction score indicates better alignment with an annotated direction.

The direction score on a dataset of interest is the average across boundary cells, as

20 $$\begin{aligned} DS = \frac{1}{|\cap _{(A, B)} \mathcal {N}_{A \rightarrow B}|} \sum \limits _{i \in \cap _{(A, B)} \mathcal {N}_{A \rightarrow B}} DS(i), \end{aligned}$$

where $\cap _{(A, B)} \mathcal {N}_{A \rightarrow B}$ is the union of boundary cells in all annotated cell-type pairs. The difference between this direction score and the original CBDir score [7] is on the scope of the average in this equation. The CBDir metric used the direction score per cell-type pair as $\sum _{i \in \mathcal {N}_{A \rightarrow B}}$ and then computed the average over pairs. However, not all cell-type pairs have the same number of cells and the result score can be unequally biased toward the performance on the small portion of neighborhood boundary cells when $|\mathcal {N}_{A \rightarrow B}|$ is small. This is problematic in terms of evaluation since a subtle performance change on a small number of cells can influence the final results. Therefore, Eq. 20 instead computes the global average over boundary cells and ensures individual cell scores contribute equally to the final result.

### Computing cell-to-cell connectivity graph

The similarity of velocity vectors of cells could model cell-to-cell connectivities. We use the connectivity graph for downstream tasks, including driver gene analysis and developmental trajectory inference.

The weight in the velocity graph, $w_{ij}$ denotes the estimated magnitude of the connection. Higher $w_{ij}$ means the future state of cell *i* is close to the current state of cell *j*. $w_{ij}$ could be computed by possible similarity measures between velocity $v_i$ and the gene expression difference $s_j - s_i$. Here, we used the cosine similarity, which is also adopted in scVelo [2], therefore,

$$\begin{aligned} w_{ij} = \frac{v^T_i(s_j - s_i)}{||v_i|| \cdot ||s_j - s_i||}. \end{aligned}$$

We compute the velocity graph on selected genes with high fitting confidence by default. Selecting these genes from the complete set of highly variable genes is conducted by filtering on continuity scores (“Continuity score as a confidence measure for velocity estimation”), correlation scores (“Correlation score”), and residuals to linear regression. The residual filter follows the convention used in scVelo [2] where they first fit linear regression between spliced and unspliced expression and select genes that have relative residuals larger than zero and smaller than 0.95.

For the visualization of the velocity plot, we adopted the same projection computation provided by exiting methods [1, 2] to project velocity as arrows onto low-dimensional embeddings, such as tsne [41] and UMAP [17]. To summarize, the transition probability $\pi _{i,j}$ from a cell *i* to possible target cell *j* is computed by the Gaussian normalized connectivity weight $w_{ij}$. Then, the velocity vector for $v_i$ in a low-dimensional space is computed by the weighted sum of $\sum _{j}\pi _{i,j} \delta _{ij}$, where $\delta _{ij}$ is the direction vector pointing from cell *i* to *j* in the low-dimensional space.

### Driver gene estimation and comparison

#### Mouse hindbrain development data

To determine functional signals in the driver genes, the top 100 genes based on positive correlation with each lineage were determined, in particular for the hindbrain developmental data from [11]. Positive correlations were used as many genes are anti-correlated between the two lineages and considering all genes would result in a similar set of drivers and functional signatures. Selecting positive genes enforces the constraint of upregulation across a developmental pseudotime. Overlap of driver genes with marker genes based on the original analysis used to annotate cell-types was performed, as well as overlap with transcription factors. Transcription factors were pulled from the manually annotated Human Transcription Factors list curated by [42] and were lifted over to mouse data using orthologous gene-matches.

Analysis of marker overlap was further extended by determining the ranking of marker genes across all tested driver genes (2000 total) for both scVelo and DeepVelo per lineage in the [11] data. The DeepVelo and scVelo predicted rankings of these marker genes for both lineages were compared, where a higher ranking of marker genes indicated a stronger signal for biologically relevant genes in the driver gene analysis. Since the entire tested driver gene lists were used, the number of genes per lineage was equivalent, and the rankings of the two lists were compared using the Mann-Whitney *U* test (or Wilcoxon rank-sum test), which is a non-parametric test for differences in sample distributions. The two-sided version of the test was used in this case, allowing either DeepVelo or scVelo to have greater or lesser rankings for relevant marker genes. The top driver genes for each lineage were also tested for functional signal using pathway enrichment analysis.

#### Pilocytic astrocytoma data

We computed the correlation between individual genes and the pseudotime of each branch. Notably, the branches are determined by the louvain clusters [43] calculated using the DeepVelo velocity graph (Additional file 1: Fig. S17). Similar to the hindbrain development data, the top 100 positively correlated driver genes were determined for each branch in each PA sample independently. Afterwards, the driver genes from each branch were tested for enrichment of functional signatures using pathway enrichment analysis.

### Pathway enrichment analysis

#### Mouse hindbrain development data

To determine functional signals in the driver gene results, pathway enrichment analysis was done using the ActivePathways R package [44]. The top 100 driver genes, based on positive correlation values with pseudotime for both the GABAergic and gliogenic lineages from the [11] data, were input into the ActivePathways gene-set enrichment analysis model. The latest Gene-Matrix-Transposed (GMT) files containing gene-set information from the Gene Ontology Molecular Function, GO Biological Process, and REACTOME databases were used [45, 46]. Pathways were labeled as being involved in “Neurogenesis,” “Developmental non-neuronal,” and “Non-specific” using manual annotation and the presence of known terms (such as “neuron projection” or “proliferation” for “Neurogenesis” and “Developmental non-neuronal,” respectively). “Non-specific” pathways indicated those that did not have immediately obvious roles in either neurogenesis or general development. To determine significant differences between pathway labeling and potential enrichment of neurogenic/development specific pathways, a two-sided Fisher’s exact test based on the hypergeometric distribution was done for the contingency table comprising of scVelo and DeepVelo pathway results and functional labels (“Neurogenesis,” “Developmental non-neuronal,” “Non-specific”) for the gliogenic and GABAergic lineages independently.

#### Pilocytic astrocytoma data

Similar to the hindbrain development analysis, the GO Molecular Function, GO Biological Process, and REACTOME databases were used [45, 46] with the ActivePathways model, but in this case, the human gene sets were used as the PA data is from human tumors. Pathway enrichment analysis was done for the two branches in each of the three PA samples based on the top 100 positively correlated driver genes. After this step, the branches were labeled as ’immunogenic’ or ’depleted’ based on the enrichment of immune-related pathways. The enrichment results were visualized using Cytoscape [47] and EnrichmentMap [48]. Unlinked and single-linked pathways were removed from the visualization at a linkage threshold of 0.5 based on the gene set similarity coefficient [47]. As many pathways in close proximity were present, groups of pathways were abstracted to higher-level names for visualization, such as “Apoptosis” for a group of apoptosis-related pathways.

### Supplementary information

**Additional file 1:** **Supplementary notes S1-S5 and Figures S1-S29.**

**Additional file 2:** **Supplementary Table S1.**

**Additional file 3:** **Supplementary Table S2.**

**Additional file 4:** **Supplementary Table S3.**

**Additional file 5:** **Supplementary Table S4.**

**Additional file 6:** **Supplementary Table S5.**

**Additional file 7:** **Supplementary Table S6.**

**Additional file 8:** **Supplementary Table S7.**

**Additional file 9:** **Supplementary Table S8.**

**Additional file 10:** **Supplementary Table S9.**

**Additional file 11.** Review history.

## Data Availability

All processed datasets are publicly available at Figshare [49] and can also be accessed via https://github.com/bowang-lab/DeepVelo/tree/main/examples. The only exception is the pilocytic astrocytoma samples from the study by Vladoiu et al. [11], which are under controlled access at the European Genome-Phenome Archive (EGA), accession EGAS00001003170 [50]. The raw data of the dentate gyrus neurogenesis, mouse hippocampus, and pancreatic endocrinogenesis are available at the Gene Expression Omnibus (GEO) repository, with accession number GSE95753 [51], GSE104323 [52], and GSE132188 [53], respectively. The mouse gastrulation [13] is available at https://github.com/MarioniLab/EmbryoTimecourse2018. The curated versions of these datasets are hosted at https://scvelo.readthedocs.io/api/#datasets. The raw BAM files for the mouse hindbrain development data are available at GEO with accession number GSE118068 [54], and the processed loom files are available at Figshare [49]. The processing steps of our custom Kallisto pipeline utilized the raw fastq files instead of the BAM files. The raw data for the Cao et al. organogenesis data are available in GEO under accession number GSE119945 [55]. The DeepVelo package, data processing code and all results reported are available at https://github.com/bowang-lab/DeepVelo and Zenodo [56]. The processed data and the DeepVelo package are released with MIT license.
