# Supplementary material for: DeepVelo: deep learning extends RNA velocity to multi-lineage systems with cell-specific kinetics
Source: Genome Biol. 2024 Jan 19;25:27. doi: 10.1186/s13059-023-03148-9 (PMC10799431; doi:10.1186/s13059-023-03148-9)
Supplement: Supplementary file 1 — Additional file 1: Supplementary notes S1-S5 and Figures S1-S29. [file 13059_2023_3148_MOESM1_ESM.pdf]

Supplementary Notes

S1 Robustness to Hyperparameters

To determine the robustness of DeepVelo's velocity predictions to model hyperparameters, a random-search over model and objective hyperparameters was performed using the Dentate Gyrus dataset. The resulting velocity estimates, overall and cell-type-wise consistency scores, and direction score were evaluated using different sampled hyperparameters. The search space over the parameters is indicated in Inline Table 1, and a total of 5000 model runs were sampled using the indicated search space. A complete summary of the results is indicated in Additional file 9: Table S8.

A more complete description of the hyperparameter names in Inline Table 1:

- **Learning rate** - The learning rate used for the optimization of the DeepVelo model, which scales the step sizes taken after each gradient descent update.
- **Optimizer** - The optimizer utilized for training the DeepVelo model. The choice of optimizer dictates the process of optimizing DeepVelo's objective and in general can influence factors such as the model being stuck in a local minima. We include three commonly used optimizers, stochastic gradient decent (SGD), RMSprop, and ADAM [1].
- **k for KNN in GNN embedding** - The GNN is applied on the nearest neighbor graph of cells. k controls the number of neighbor nodes that each cell is connected to on the graph.
- **k for KNN in *t+1* GNN embedding** - The number of neighbors that are considered as potential future cell states at *t+1*. This is the size of $\mathcal{N}_i$ in Methods Eq.12 and Eq.13.
- **Number and size of neural network layers** - indicates the number and size of the hidden layers used in the GNN model. Theoretically, additional neural network layers can learn more abstract/higher-level concepts.
- **Pearson correlation coefficient scaling** - The coefficient to use for scaling the Pearson correlation term with respect to the Maximum Likelihood Estimation term (see Online Methods). Indicates the weight put on the Pearson correlation term.
- **Preprocessing - number of highly variable genes** - the number of highly variable genes to subset the spliced and unspliced counts to before further processing of the data.
- **Preprocessing - number of neighbors for expression smoothening** - the number of nearest neighbors to use for smoothening the expression values for RNA velocity estimation.
- **Preprocessing - number of principal components for expression smoothening** - the number of principal components to use for the reduction utilized to calculate nearest neighbors for expression smoothening.

As indicated by Additional file 1: Fig. S19-S27, cell-type wise and overall consistency scores are robust to all hyperparameters tested and the only differences in results arose when considering the optimizer and learning rate utilized to train the DeepVelo model (Additional file 1: Fig. S19, S20). This is an expected result, as both the choice of optimizer and learning rate can affect the path of gradient descent and the resulting

Table 1 Search space of hyperparameters tested

Hyperparameter	Sample space
Learning rate	[0.0001, 0.001, 0.01, 0.1]
Optimizer	[Adam, SGD, RMSprop]
k for KNN in GNN embedding (topG)	[5, 10, 20, 30, 40, 50]
k for KNN in *t+1* GNN embedding (topC)	[5, 10, 20, 30, 40, 50]
Number and size of neural network layers	[[32, 32], [64, 64], [128, 128], [256, 256], [512, 512], [64, 64, 64]]
Pearson correlation coefficient scaling	$U \sim [1, 100]$
Preprocessing - number of highly variable genes	[500, 1000, 2000, 2500, 5000]
Preprocessing - number of neighbors for expression smoothening	[5, 10, 15, 20, 30, 40, 50]
Preprocessing - number of principal components for expression smoothening	[10, 20, 30, 40, 50]

final parameter set utilized to obtain the velocity estimates. Although the stochastic gradient descent (SGD) optimizer led to notable increases in consistency scores (Additional file 1: Fig. S19), it led to worsening performance in the direction score, meaning it led to smoother estimates but the wrong direction. Given this result, we opt to continue using the Adam optimizer. The other notable result is that increasing the number of nearest-neighbors for smoothening the initial spliced/unspliced counts (number of preprocessing neighbors - Additional file 1: Fig. S26) led to increased scores for both consistency and direction scores. Given this result, we will opt to increase the default number of neighbors for the next update of the DeepVelo python package.

The results based on learning rate indicate that DeepVelo is also robust to overfitting. Varying the learning rate between 0.1-0.0001, which is a very large range, led to only marginal changes in cell-type wise and overall consistency scores (Additional file 1: Fig. S20). Direction scores did vary by percentage much more based on learning rate, but most of the variance from the best parameter setting (0.001) was from a very high learning rate (0.1) or a very low learning rate (0.0001) (Additional file 1: Fig. S20). Within a more reasonable learning rate range of 0.001-0.01, the DeepVelo direction scores are stable, indicating that this is not a highly sensitive hyperparameter for the model. We also additionally test 100 runs with the best learning rate 0.001 and Adam optimizer, and record the average scale of velocities. We found the relative change of scale between runs is small within 10%, ranging from 0.084 to 0.094.

Lastly, we sought to determine if DeepVelo's choice of graph convolution layers for learning an embedding corresponding to the determined kinetics rates is appropriate. The DeepVelo framework can work with any type of forward propagation layer, including fully-connected linear layers. We compared DeepVelo's implementation of graph convolution layers (GCN) with a version that used fully-connected layers (FFNet) (Additional file 10: Table S9). In terms of overall consistency, there were negligible differences between the GCN and FFNet implementations across datasets. The FFNet resulted in higher cell-type wise consistency scores (Additional file 10: Table S9), but this may be attributed to optimization differences between the two types of layers. For the direction score, which we consider the most important metric, there were negligible differences for 3/5 datasets, but for the dentate gyrus neurogenesis and

large-scale hippocampus datasets, the GCN layer had substantially higher scores, by +0.11 and +0.35, respectively (Additional file 10: Table S9). This result is likely due to the implicit regularization that the GCN layers offer, which prevents overfitting to an incorrectly inferred direction [2]. Given this result, the GCN layer is the most appropriate for the DeepVelo model implementation.

Overall, the hyperparameter tuning results revealed that DeepVelo is robust to key preprocessing, model-based, and optimization-based hyperparameters, and the DeepVelo model, objective, and optimization procedure are insensitive to these changes.

S2 Theoretical analysis of the continuity assumption

We show in this section that any system that satisfies the steady-state assumption or the dynamical model of scVelo [3] will satisfy the continuity assumption in Eq.7. In other words, the continuity assumption is more generalized than previous steady-state assumptions.

We start from analyzing the temporal spacing between data points in scRNA-seq data to assist the derivation for the error bound of our estimates. Suppose a biological system of interest undergoes a cellular development process ranging from development time 0 to T. A single-cell RNA sequencing experiment conducted on the cells of this system can be considered a sampling process that observes the spliced (and unspliced) mRNA expression s_i of individual cells, $i = \{1, 2, 3, .., n\}$. n is the number of cells captured in the experiment, Ω. s_i is an observed expression of a spliced mRNA g in cell i. We omit the subscript g here and later in this section for simplicity. The development time of each cell is denoted as t_i, $0 < t_i < T$. For the following analysis without loss of generality, we naturally require each sub-period in $(0, T)$ must contain a sufficient portion of sequenced cells, since the faithfulness of all learning methods require the existence of enough data. Without other priors and for simplicity, we then assume the sequencing samples are uniformly sampled in the time range $(0, T)$, i.e. $t_i \overset{\text{iid}}{\sim} U(0, T)$. For a specific data point t_i, we are interested in the distance/spacing to the nearest sample with time $> t_i$. The distribution of this nearest distance takes the form of **beta distribution**, $Beta(1, n)$. We provide the proof in Additional file 1: Note S2.1. For a time range $(0, 1)$, this has the probability density function (PDF) and cumulative distribution function (CDF) as follows,

$$
\begin{aligned}
f(x) &= n(1-x)^{n-1} \\
F(x) &= 1 - (1-x)^n, \quad 0 < x < 1.
\end{aligned}
$$

For time range $(0, T)$, the PDF and CDF are

$$
\begin{aligned}
f(x) &= \frac{n}{T}(1 - \frac{x}{T})^{n-1} \\
F(x) &= 1 - \left(1 - \frac{x}{T}\right)^n, \quad 0 < x < T.
\end{aligned} \tag{21}
$$

This is a derived distribution by scaling the original Beta distribution to $(0, T)$. The expectation is $\frac{T}{n+1}$.

Remark 1

The uniform distribution of samples on the time range $(0, T)$ is required in the above derivation. First, it is reasonable to assume an equal probability of observation on the time points in the range of interest when there is no other prior. Secondly, the core conclusion we need for later analysis from the above derivation is the CDF $F(x)$ of the nearest distance distribution. So given other sampling priors, any sampling distribution that can generate a nearest distance CDF $F_1(x) >= F(x), 0 < x < T$, will have a tighter error bound than the ones we show later in Corollary 1 and 2. In other words, we view the uniform distribution assumption merely as a base requirement for the "density" of cells in the data. And since sufficient data is always required for any machine learning method, we believe the assumption we made here is well generalized to any reasonable application scenarios.

We show in the following analysis that the above distribution of the nearest spacing gives an error bound for the future neighbors in the continuity assumption, i.e. the learning steps Eq.7-11. We name the function for the mRNA expression in the sequenced time period $(0, T)$ as the gene dynamic function, $f_{dyn}(t)$. In other words, $s_i = f_{dyn}(t_i)$ for a sequenced data point $s_i \in \Omega$. We are interested in its future cell state at a small time step Δt, which is $f_{dyn}(t_i + \Delta t)$. This point at $t_i + \Delta t$ may not be observed. To approximate it, the continuity assumption, Eq.7, uses the expectation of close future neighbors to estimate the $f_{dyn}(t_i + \Delta t)$. The optimal strategy of selecting future neighbors for this selection is dependent on the varying form of f_{dyn}. We denote the estimation error of this optimal strategy ϵ. We set up another surrogate strategy using the sample at the nearest spacing x that follows the distribution Eq.21. It will have a larger error, $\epsilon_x \geq \epsilon$. This error ϵ_x comes from the learning step Eq.11 where the velocity estimate is optimized towards the differentiated cells. The strategy of selecting the sample at the nearest spacing as future neighbors can be rewritten in the notation we used here in this section. The velocity estimate $\tilde{v}_i$ is,

$$\begin{aligned} \tilde{v}_i &= \frac{\text{(expression at the nearest spacing x)} - s_i}{\Delta t + x} \\ &= \frac{f_{dyn}(t_i + \Delta t + x) - f_{dyn}(t_i)}{\Delta t + x} \end{aligned} \tag{22}$$

The actual velocity is defined as the derivative of f_{dyn},

$$v_i = \frac{ds_t}{dt} = f'_{dyn}(t_i) \tag{23}$$

So the error of this strategy is $\epsilon_x = |\tilde{v}_i - v_i|$. Using Taylor expansion at time t_i, we can approximate the function $f_{dyn}(t_i + \Delta t + x)$ as follows:

$$f_{dyn}(t_i + \Delta t + x) = \sum_{k=0}^{\infty} \frac{f_{dyn}^{(k)}(t_i)}{k!} (\Delta t + x)^k$$

Remark 2

The existence of high-order derivatives of $f^{(k)}_{dyn}(t)$ is required in the expansion, which is a reasonable precondition for gene dynamics. There are two reasons for this - (1) Transcription is a smooth process happening in the physical world. The derivatives of mRNA abundance are determined by all the factors and co-factors participating in the process, such as RNA polymerase. A non-differentiable f_{dyn} will indicate a sudden change in the abundance of molecules in an infinitely small time step, which is not possible. (2) This precondition is also guaranteed in existing approaches since previous velocity estimation methods all assume constant kinetic rates as in Eq.2. The function in the equation is infinitely differentiable with respect to t.

Given the expansion of $f_{dyn}(t + \Delta t + x)$, the difference between $\tilde{v}_i$ and v_i is,

$$\begin{aligned}
\tilde{v}_i - v_i &= \frac{f_{dyn}(t_i + \Delta t + x) - f_{dyn}(t_i)}{\Delta t + x} - f'_{dyn}(t_i) \\
&= \frac{\sum_{k=0}^{\infty} \frac{f^{(k)}_{dyn}(t_i)}{k!}(\Delta t + x)^k - f_{dyn}(t_i)}{\Delta t + x} - f'_{dyn}(t_i) \\
&= \frac{\sum_{k=1}^{\infty} \frac{f^{(k)}_{dyn}(t_i)}{k!}(\Delta t + x)^k}{\Delta t + x} - f'_{dyn}(t_i) \\
&= \frac{(\Delta t + x) f'_{dyn}(t_i) + \sum_{k=2}^{\infty} \frac{f^{(k)}_{dyn}(t_i)}{k!}(\Delta t + x)^k}{\Delta t + x} - f'_{dyn}(t_i) \\
&= \frac{\sum_{k=2}^{\infty} \frac{f^{(k)}_{dyn}(t_i)}{k!}(\Delta t + x)^k}{\Delta t + x} \\
&= \sum_{k=2}^{\infty} \frac{f^{(k)}_{dyn}(t_i)}{k!}(\Delta t + x)^{(k-1)}
\end{aligned} \tag{24}$$

Therefore, the difference $\tilde{v}_i - v_i$ is dependent on the choice of Δt and the value of nearest spacing x. The Δt controls the precision of the numerical derivative. In practice, it is practical to set it to the expected time gap between two samples. This is $\Delta t = \frac{T}{n+1}$ for uniform samples in $(0, T)$. x is the value of the random variable X that has the CDF as Eq.21. Therefore, by probability $1 - \left(1 - \frac{x}{T}\right)^n$, the error bound of the optimal estimator is,

$$\epsilon \leq \epsilon_x = |\tilde{v}_i - v_i| \leq \left| \sum_{k=2}^{\infty} \frac{f^{(k)}_{dyn}(t_i)}{k!} (\frac{T}{n+1} + x)^{(k-1)} \right| \tag{25}$$

This equation indicates that on more "dense" data with smaller x, we can have more confidence about a smaller error bound by a certain probability. We are particularly interested in two cases, (1) a relaxed error bound when x has asymptotic scale $O(\frac{1}{\sqrt{n}})$ and (2) a tighter error bound when x is asymptotically $O(\frac{1}{n})$.

Firstly, when $x \leq \frac{T}{\sqrt{n}} = O(\frac{1}{\sqrt{n}})$, we have the following result based on Eq.25. By probability $p_{1/2} = 1 - \left(1 - \frac{1}{\sqrt{n}}\right)^n$, the error bound is,

$$\begin{aligned} \epsilon &\leq \left| \sum_{k=2}^{\infty} \frac{f_{dyn}^{(k)}(t_i)}{k!} (\frac{T}{n+1} + \frac{T}{\sqrt{n}})^{(k-1)} \right| \\ &< \left| \sum_{k=2}^{\infty} \frac{f_{dyn}^{(k)}(t_i)}{k!} (\frac{2T}{\sqrt{n}})^{(k-1)} \right| \end{aligned} \tag{26}$$

The error bond decreases asymptotically when n grows. When n approaching infinity, $\lim_{n\to\infty} p_{1/2} = 1$ and

$$\lim_{n\to\infty} \epsilon \leq \lim_{n\to\infty} \left| \sum_{k=2}^{\infty} \frac{f_{dyn}^{(k)}(t_i)}{k!} (\frac{2T}{\sqrt{n}})^{(k-1)} \right| = 0.$$

We summarize this result in the following corollary.

Corollary 1

If unlimited samples (cells) are present, the error of the optimal estimator using continuity assumption converges to 0.

This corollary only requires the uniform and differentiable preconditions we discussed in Remarks 1 and 2. Since we already showed in those remarks that these preconditions are reasonable for all scRNA-seq data where velocity is going to be estimated. Therefore, Corallary 1 indicates the convergence of the optimal estimator of the continuity assumption on RNA velocity applications.

Next, to discuss the more realistic situation where a finite n number of cells are observed in the data, we examine the case when x is $O(\frac{1}{n})$ for a tighter error bound. Here without loss of generality, we examine the case $x \leq \frac{10T}{n}$. Based on Eq.25, by probability $p_1 = 1 - \left(1 - \frac{10}{n}\right)^n$, the error is bounded as,

$$\begin{aligned} \epsilon &\leq \left| \sum_{k=2}^{\infty} \frac{f_{dyn}^{(k)}(t_i)}{k!} (\frac{T}{n+1} + \frac{10T}{n})^{(k-1)} \right| \\ &< \left| \sum_{k=2}^{\infty} \frac{f_{dyn}^{(k)}(t_i)}{k!} (\frac{11T}{n})^{(k-1)} \right| \\ &< \left| \frac{11}{2} \cdot \frac{f''_{dyn}(t_i)T}{n} \right| + O(\frac{1}{n^2}) \end{aligned} \tag{27}$$

Corollary 2

When estimating the RNA velocity on a dataset with n sequenced cells, by a probability at least $p_1 = 1 - \left(1 - \frac{10}{n}\right)^n$, the asymptotic error of the optimal estimator using continuity assumption is bounded by $O(\frac{1}{n})$.

The probability p_1 in Corollary 2 is rather large and likely to be satisfied in any realistic setting where more than 1000 cells are sequenced, $n > 1000$. This is because p_1 has a limit at $1 - e^{-10} = 1 - 4.5 * 10^{-5}$, and it quickly approximates the limit when n grows. For example, this probability is $p_1 = 0.9996$ when $n = 1000$.

Corollary 2 indicates the decreasing of error by the speed of $O(\frac{1}{n})$, again, for any differentiable gene dynamic function. This broadens the scope of application of the previous scVelo dynamical or velocyto approaches, where constant kinetic rates are further required. In fact, even in the special case where the gene dynamic function has constant kinetics rates as in Eq.2, the EM algorithm used in scVelo has been reported with asymptotic error decreasing at $O(\frac{1}{\sqrt{n}})$ (Equation 59 and 63 in [4]). Therefore, the error decreasing speed of the optimal estimator at $O(\frac{1}{n})$ is faster.

To summarize, Corollary 1 proves the convergence of the optimal estimator using the continuity assumption in the setting of unlimited observations. In the more practical setting of finite n observations, Corollary 2 shows the faster decrease of the asymptotic error at the speed of $O(\frac{1}{n})$ by probability close to 1. This is generalized for all meaningful gene dynamics that are differentiable, and is faster than previous approaches even in the special constant-rate conditions those approaches assume. It is worth noting that (1) theoretically, although this error bound is derived for the optimal estimator, the contribution in general is clearly presented: the continuity can be readily used as a fast and reliable learning objective generalized to all differentiable gene dynamics. (2) Empirically, although the analytical form of the optimal estimator is unknown, we build the feasible solution of DeepVelo using deep learning and the continuity assumption (Online Methods), and show the advantages on varying experiments in this work.

S2.1 Probability of the temporal distance to the nearest data sample

Let X be the distance from a point uniformly sampled from the interval (0, 1) to its nearest neighbor that has a value larger than the point, and let n be the number of points sampled uniformly at random from the same interval.

To find the PDF of X, we consider the probability that the nearest neighbor of the sampled point is at a distance between x and $x + dx$. This probability is equal to the probability that there is no point within a distance x of the sampled point, multiplied by the probability that there is a point within a distance dx of the sampled point. The probability that there is no point within a distance x of the sampled point is given by the probability that all n points are at least x away from the sampled point. This probability is:

$$P(\text{no point within a distance } x) = (1 - x)^n \tag{28}$$

The probability that there is a point within a distance dx of the sampled point is given by the density of points, which is 1. So the probability that the nearest neighbor is between x and $x + dx$ is:

$$P(X = x)dx = n(1 - x)^{n-1}dx \tag{29}$$

Therefore, the PDF of X is:

$$f(x) = n(1 - x)^{n-1} \tag{30}$$

for $0 < x < 1$. To find the CDF of X, we integrate the PDF from 0 to x:

$$\begin{aligned} F(x) &= \int_0^x f(t)dt \\ &= \int_0^x n(1-t)^{n-1}dt \\ &= n\int_0^x (1-t)^{n-1}dt \\ &= -n\int_{1-x}^1 u^{n-1}du \qquad \text{(substitute } u = 1 - t\text{)} \\ &= -n\left[\frac{u^n}{n}\right]_{1-x}^1 \\ &= -\left[(1-1)^n - (1-(1-x))^n\right] \\ &= 1 - (1-x)^n \end{aligned} \tag{31}$$

Notably, the PDF and CDF of this distribution take the form of the Beta distribution $B(1, n)$.

S3 Estimating velocities on mouse gastrulation data with MURK genes

We tested DeepVelo on the challenging mouse gastrulation data from [5]. This dataset is reported to have challenging dynamics by [6], and the authors identified multiple rate kinetic (MURK) genes that undergo complex dynamics, such as transcriptional bursts, which violate the steady-state assumption. We first confirmed that neither the dynamical nor steady-state models estimate the correct directions based on the ground-truth lineage (Additional file 1: Fig. S18f,g). Conversely, both approaches generate opposite directions from erythroid cells to progenitor cells.

DeepVelo is able to accurately estimate the RNA velocity directions aligned with actual erythrocyte differentiation, and demonstrates the correct trend of pseudotime (Additional file 1: Fig. S18a,b). The quantitative metrics show significantly higher scores by DeepVelo reflecting the visual findings (Additional file 1: Fig. S1). We found setting Pearson correlation heuristic term (Eq.14) only in the first 10 epochs helps improve the model performance. We discuss this influence of Pearson heuristics in Additional file 1: Note S4. We also show two typical MURK genes identified by [6]. The

DeepVelo estimates of RNA velocity on these genes individually point to the correct direction toward the differentiated cell types (Additional file 1: Fig. S18c,d).

The results on the mouse gastrulation data demonstrate that DeepVelo is more capable than previous methods for velocity estimation on datasets with genes that have multiple kinetic rates. This confirms our previous theoretical analysis that the continuity assumption is applicable for any differentiable dynamics, including the transcriptional bursts of MURK genes which previous techniques fail to model correctly.

S4 Pearson correlation heuristics and improving estimation reliability with gene-wise confidence filtering

As stated in Section 1.3.2, the Pearson correlation term in Eq.14 helps in guiding the direction for finding future neighbors. This term comes from the differential equation Eq.1 of RNA velocity, where the two terms of unspliced and spliced expression have coefficients of opposite signs. When the change of unspliced/spliced expression is much larger than the change of kinetic rates, beta/gamma, then the positive correlation of RNA velocity to unspliced counts and negative correlation to spliced counts can be directly concluded from Eq.1. This is applicable globally across most cells, where expression values usually do change dramatically in orders of magnitude (Fig. 2d,e as examples). It is worth pointing out that this pattern should be applicable only in general but is not guaranteed for all genes. In practice, we find applying the heuristics globally across cells provides strong gradients at the beginning of training to break the symmetry of the forward and backward continuity objectives in Eq.14. After the symmetry is broken fast by the Pearson heuristic, the learning via continuity can further refine the dynamics with correct directions to future neighboring cells.

To validate the above claims about the heuristic role of the Pearson correlation and show that it is not needed as a central objective for learning, we show the distribution of the Pearson correlation between the recovered velocity and the (un)spliced expression of individual genes in the Dentate Gyrus dataset (Additional file 1: Fig. S28a). We found more than 95% of the genes are concordant with the Pearson correlation heuristic with a combined correlation score greater than 0. On the other hand, we do find the genes with the highest velocity confidence scores (meaning best matching the continuity assumption in Eq.7) (Additional file 1: Fig. S28b), do not necessarily have high combined correlation scores. For example, *Ppp3ca* and *Tmsb10* have high confidence scores (among the top 10) (Additional file 1: Fig. S28d), but have relatively low correlation scores at 0.14 (smaller than 95% of genes). These findings validate our design that the Pearson correlation works to break the symmetry, while the actual learning should be more optimized towards the continuity assumption.

Although the Pearson correlation heuristic is not designed to be the focal point of the learning objective, we find it works reliably to indicate unclear gene dynamics when the correlation score is extremely low. In Additional file 1: Fig. S28c, we show the 10 genes with the smallest correlation scores. The low correlation scores reflect various pathological cases for using the gene dynamics to estimate RNA velocity, including (1) low spliced and unspliced expression captured in data - *Arhgap10*, *Kat2b*, *Myo1h*, *Sugp1*, (2) unexpected large separation between cell groups - *Fam101b*, *Polr3h*,

Sirt2 (3) celltypes completely inseparable based on expression - *Arhgap10*, *Zx2hc1a*. In practice, we set up the gene filtering step based on a combination of correlation scores, confidence scores, and a commonly used residue measurement (Online method - 1.9). The default threshold for the lowest correlation score to be included is set at 0 (green line in Additional file 1: Fig. S28a), and the default threshold for the confidence score is set at the 5th percentile value (red line in Additional file 1: Fig. S28b). We observe significant performance improvement by applying the filtering step before computing the velocity graph (Online method - 1.9) for further visualization and analysis.

S5 Tracking the ordering of cellular development using DeepVelo and diffusion pseudotime

The RNA velocity estimated by DeepVelo can also be used to improve the prediction of pseudotime for cell states across a developmental trajectory. We first compute the velocity connectivity graph to represent cell-cell relationships and use this graph as the basis to compute a diffusion pseudotime [7] mapping (Online Methods - 1.9). We compare the pseudotime estimates (Additional file 1: Fig. S10a) using DeepVelo with the latent time (Additional file 1: Fig. S10c) estimates by the dynamical model from scVelo on a scRNA-seq dataset of pancreatic endocrinogenesis with ground-truth temporal measurements. For the velocity plots, DeepVelo successfully demonstrates the main structure of endocrine progenitors developing into terminal cell-types - alpha, beta and delta (Additional file 1: Fig. S10b) with more consistent velocities (Mann-Whitney U Two-Sided Test $p < 1.0 \times 10^{-300}$, $n = 3696$ for both groups, Additional file 1: Fig. S10e). For pseudotime comparison, both methods provide accurate predictions. Notably, DeepVelo more faithfully preserves the time ordering between the terminal states of Alpha and Beta cells (Additional file 1: Fig. S10a), where the Alpha cells are developed earlier at E12.5 and the Beta cells appear later at E15.5 (Additional file 1: Fig. S10f,g).

References

[1] Kingma DP, Ba J. Adam: A method for stochastic optimization. arXiv preprint arXiv:14126980. 2014;.

[2] Nt H, Maehara T. Revisiting graph neural networks: All we have is low-pass filters. arXiv preprint arXiv:190509550. 2019;.

[3] Bergen V, Lange M, Peidli S, Wolf FA, Theis FJ. Generalizing RNA velocity to transient cell states through dynamical modeling. Nature biotechnology. 2020;38(12):1408–1414.

[4] Balakrishnan S, Wainwright MJ, Yu B. Statistical guarantees for the EM algorithm: From population to sample-based analysis. The Annals of Statistics. 2017;45(1):77 – 120. https://doi.org/10.1214/16-AOS1435.

[5] Pijuan-Sala B, Griffiths JA, Guibentif C, Hiscock TW, Jawaid W, Calero-Nieto FJ, et al. A single-cell molecular map of mouse gastrulation and early organogenesis.

Nature. 2019;566(7745):490–495.

[6] Barile M, Imaz-Rosshandler I, Inzani I, Ghazanfar S, Nichols J, Marioni JC, et al. Coordinated changes in gene expression kinetics underlie both mouse and human erythroid maturation. Genome biology. 2021;22(1):1–22.

[7] Haghverdi L, Büttner M, Wolf FA, Buettner F, Theis FJ. Diffusion pseudotime robustly reconstructs lineage branching. Nature methods. 2016;13(10):845–848.

Supplementary Figures

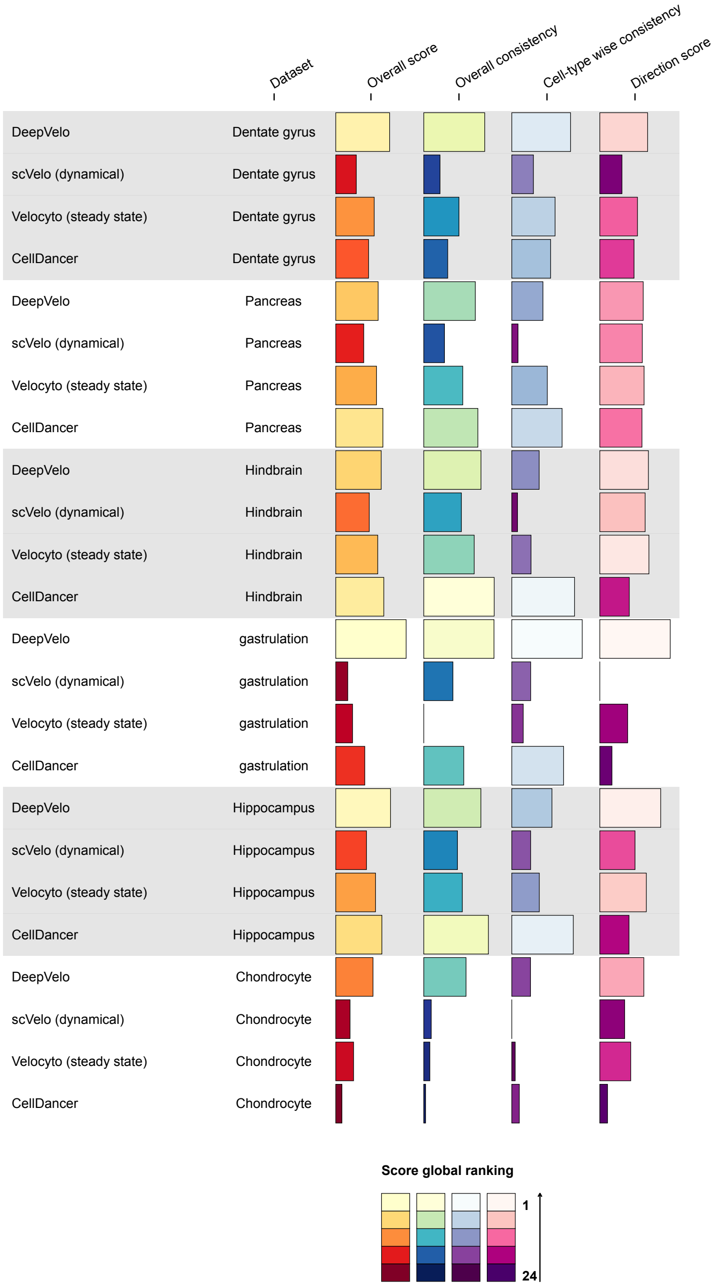

Fig. S1 Summary of evaluation results for DeepVelo and previous techniques on all datasets. The overall score considers a weighting of ($0.5 \cdot$ Direction score $+ 0.25 \cdot$ Cell-type wise consistency $+ 0.25 \cdot$ Overall consistency). The global ranking color represents the ranking for a given metric across all datasets and methods. DeepVelo demonstrates the highest overall scores on all datasets.

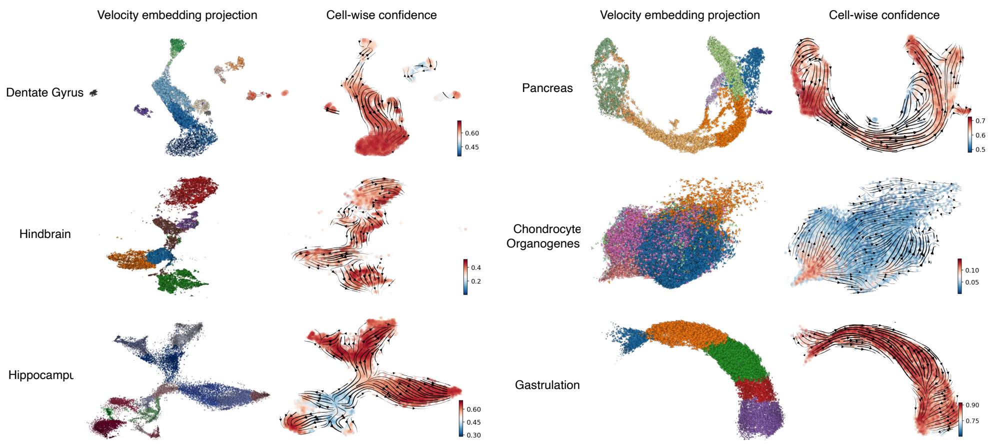

Fig. S2 Velocity plot and cell-wise confidence on all experiment datasets

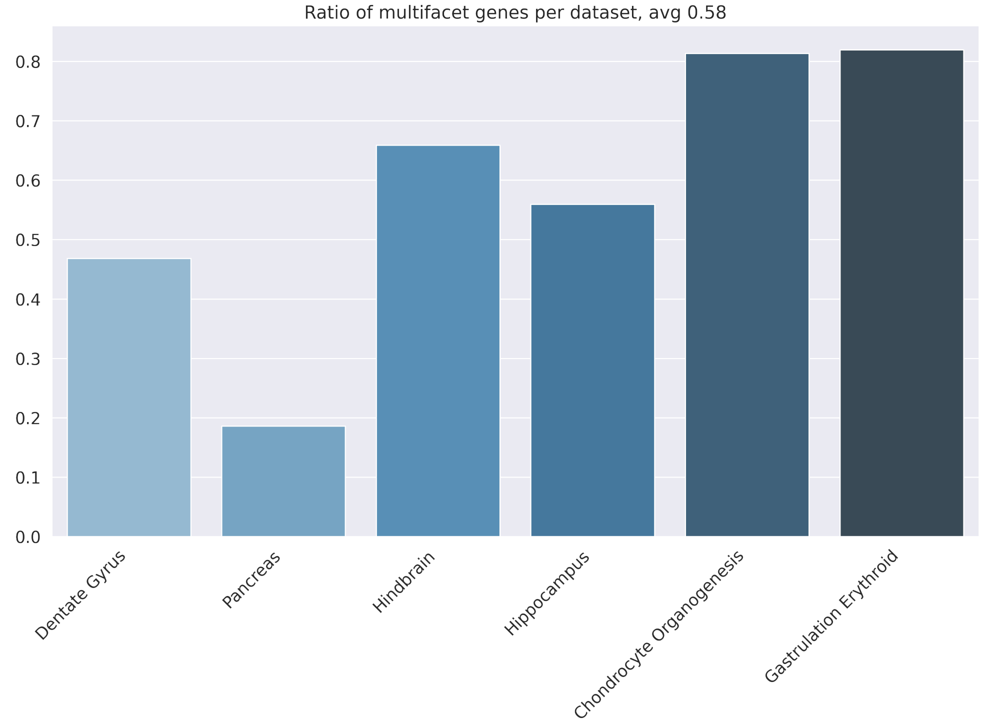

Fig. S3 Ratio of genes with multi-faceted kinetics across datasets. On all of the benchmarked datasets, the average ratio of genes with multi-faceted kinetics is 0.58. This high ratio of multifaceted gene dynamics on typical scRNA-seq data indicates the necessity of using approaches like DeepVelo, that can generalize to complex dynamics. This ratio is computed by using the official API `differential_kinetic_test` from the scVelo package on individual genes. This function tests whether the "mono-faceted" scVelo dynamical model can recover the gene dynamics with high likelihood. If not, the gene will be marked as having multiple kinetics, i.e. multifaceted.

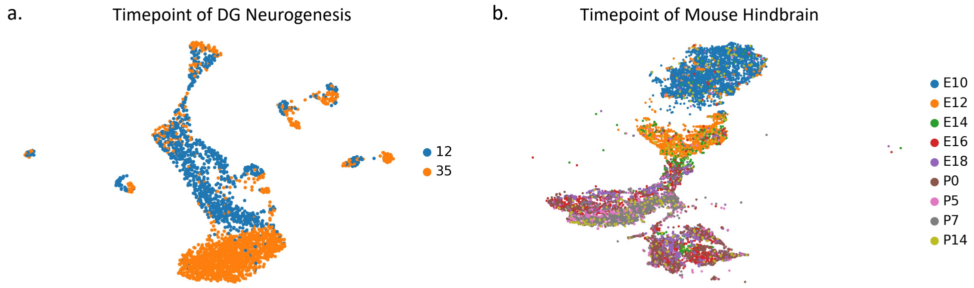

Fig. S4 The developmental time points of sequenced cells in dentate gyrus neurogenesis (a) and mouse hindbrain development (b) datasets.

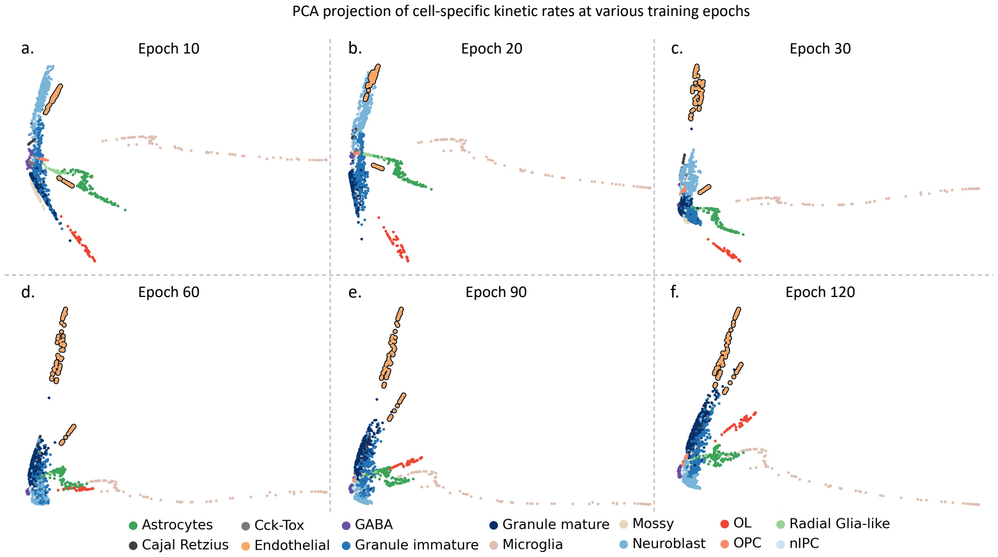

Fig. S5 The PCA projection of cell-specific kinetic rates at various training epochs. (a-f) Scatter plot of the first two PCA dimensions at training epochs 10, 20, 30, 60, 90, 120. DeepVelo learns to predict similar kinetic rates for cells of same cell-type. For example, the kinetic rates of Endothelial cells (outlined) are gradually clustered together and are located away from the unrelated granule lineage.

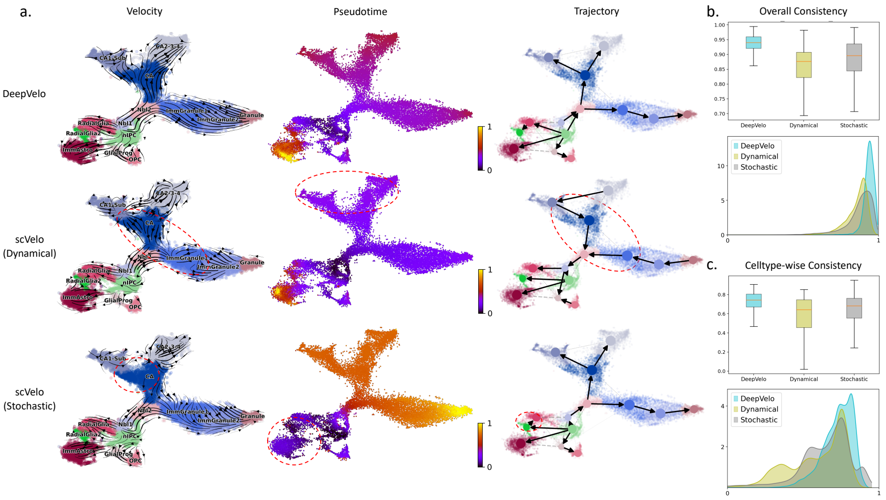

Fig. S6 Comparison of three velocity methods on large-scale hippocampus data. (a) The velocity plot, pseudotime and trajectory inference of DeepVelo, scVelo dynamical model and scVelo stochastic mode, respectively. We highlighted observed incorrect predictions of compared methods in red circles. (b, c) The overall consistency score and cell-type-wise consistency score.

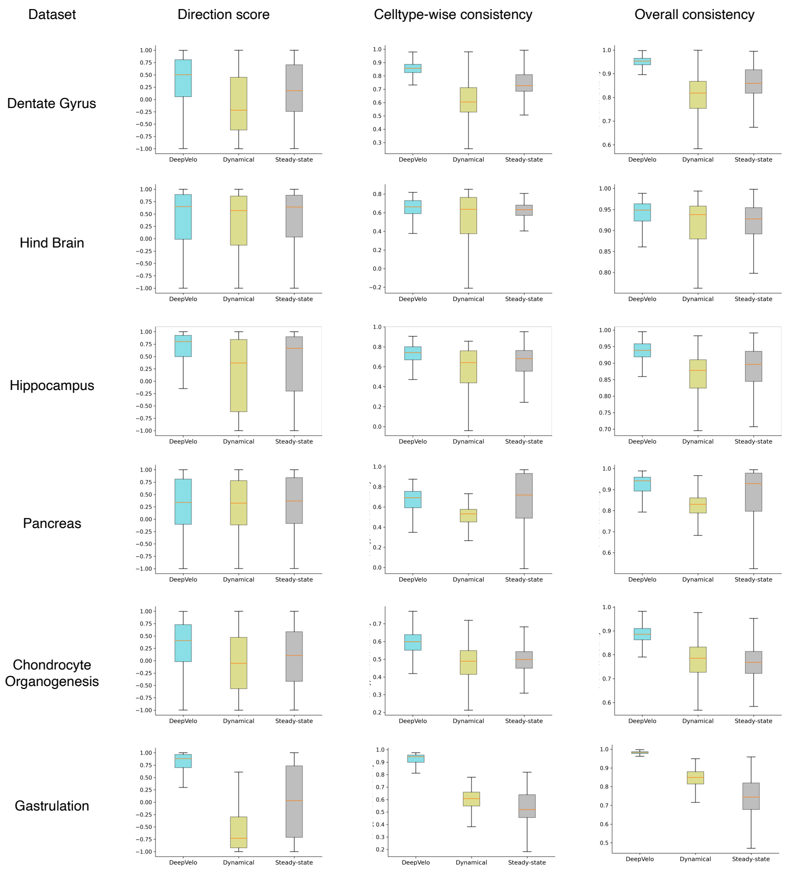

Fig. S7 The direction score, cell-type-wise consistency, and overall consistency across cells in each dataset.

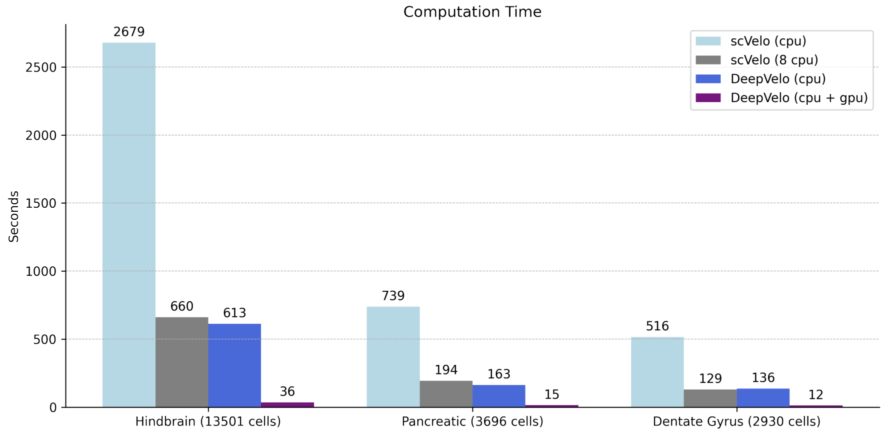

Fig. S8 Computational efficiency comparison of scVelo and DeepVelo across datasets. Using the same CPU device$^{(*)}$, DeepVelo had a 4 fold acceleration compared to the dynamical model. Using GPUs, DeepVelo can complete training and estimation for over 13,000 cells in 36 seconds. Generally the GPU-accelerated DeepVelo is 10-20 times faster than the accelerated dynamical model (8 CPUs). $(*)$ The DeepVelo(CPU) uses the pytorch package, which automatically utilizes 8 CPUs for the gradient optimization step. For all other computations, the DeepVelo(CPU) runs on single CPU.

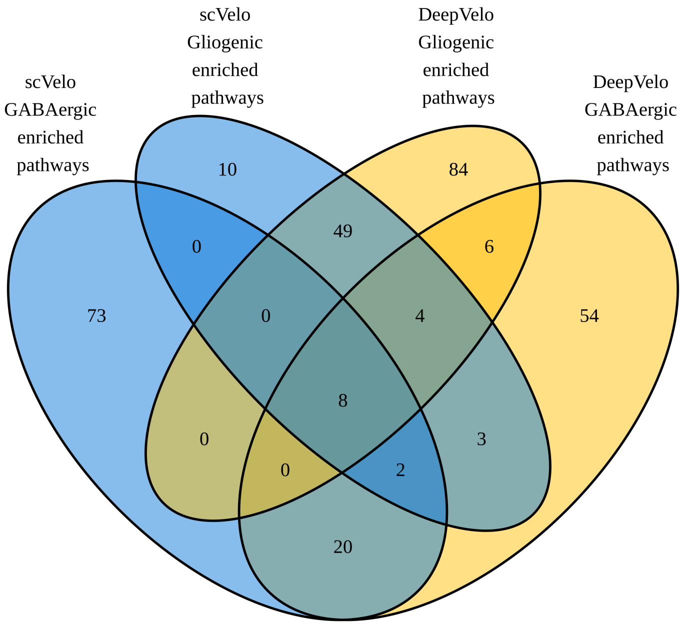

Fig. S9 Full pathway enrichment analysis results overlap. Overlap of scVelo and DeepVelo pathway enrichment analysis results, between methods, for the top 100 GABAergic and gliogenic driver genes.

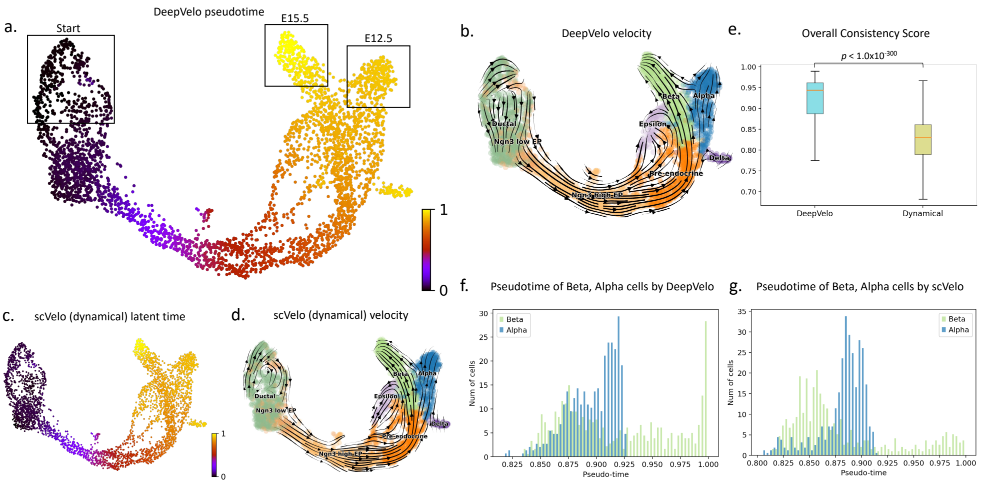

Fig. S10 Velocity and pseudotime plots for pancreatic endocrinogenesis. (a) The pseudotime prediction from DeepVelo accurately assigns alpha and beta cells to accurate developmental time points. Particularly, the progenitor cell cluster is correctly located at the upper left quadrant of the UMAP projection. The difference between the terminal alpha and beta cells is well captured, where alpha cells were developed earlier at E12.5 and beta cells appeared later until E15.5. (b) Velocity values derived from DeepVelo are projected onto the UMAP-based embedding and visualized. DeepVelo successfully captures the main structure of endocrine progenitors developing into the terminal cell-types of alpha, beta and delta cells. (c),(d) For comparison, the latent time and velocity computed by the dynamical model from scVelo. (e) Distribution of the overall RNA velocity consistency scores for DeepVelo and scVelo. (f,g) The histogram of pseudotime predictions for beta and alpha cells, by DeepVelo and the scVelo dynamical model, respectively. Beta cells are expected to have a larger percentage of cells with higher pseudotime values, which is true of the DeepVelo predicted values.

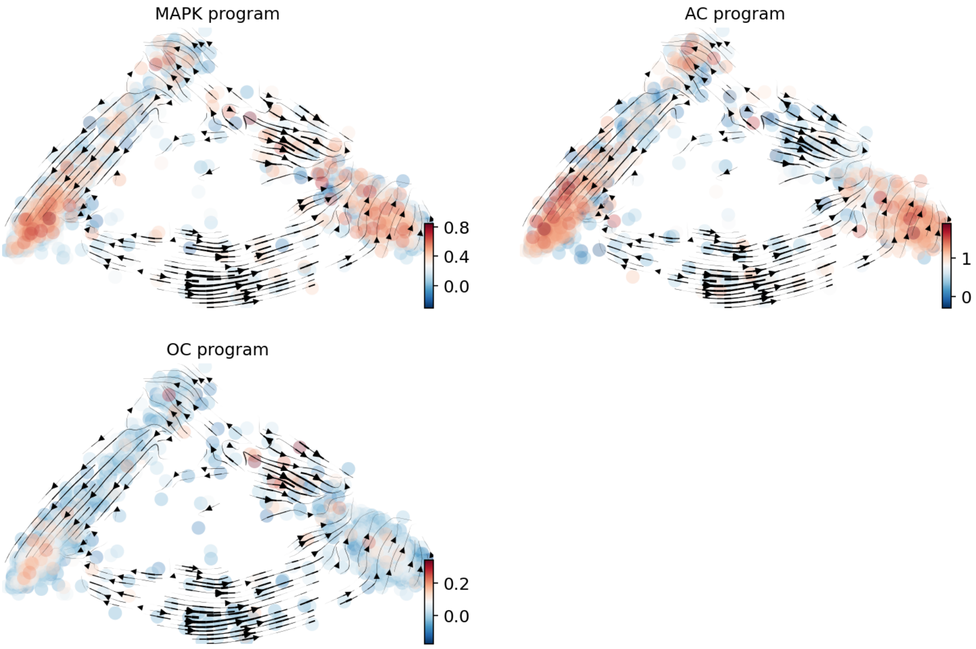

Fig. S11 MAPK, astrocyte, and oligodendrocyte module scores for PA sample 1. The signatures for the indicated pathways were determined based on the curated list of markers corresponding to each from Reitman et al.

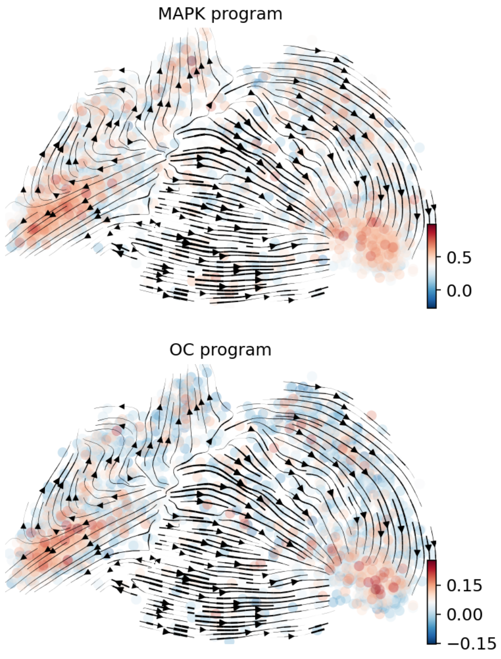

Fig. S12 MAPK, astrocyte, and oligodendrocyte module scores for PA sample 2. The signatures for the indicated pathways were determined based on the curated list of markers corresponding to each from Reitman et al.

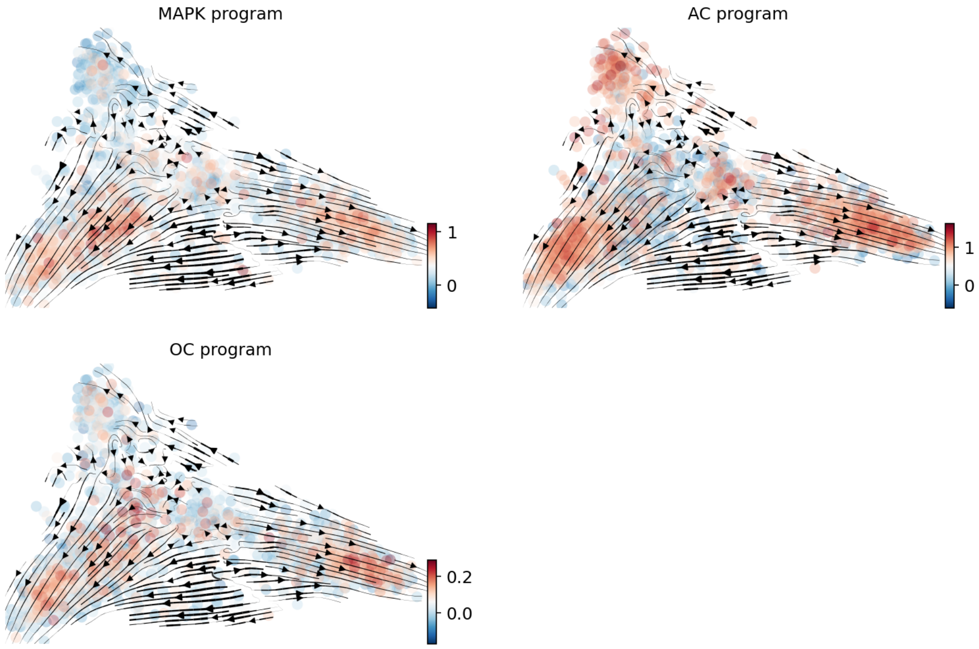

Fig. S13 MAPK, astrocyte, and oligodendrocyte module scores for PA sample 3. The signatures for the indicated pathways were determined based on the curated list of markers corresponding to each from Reitman et al.

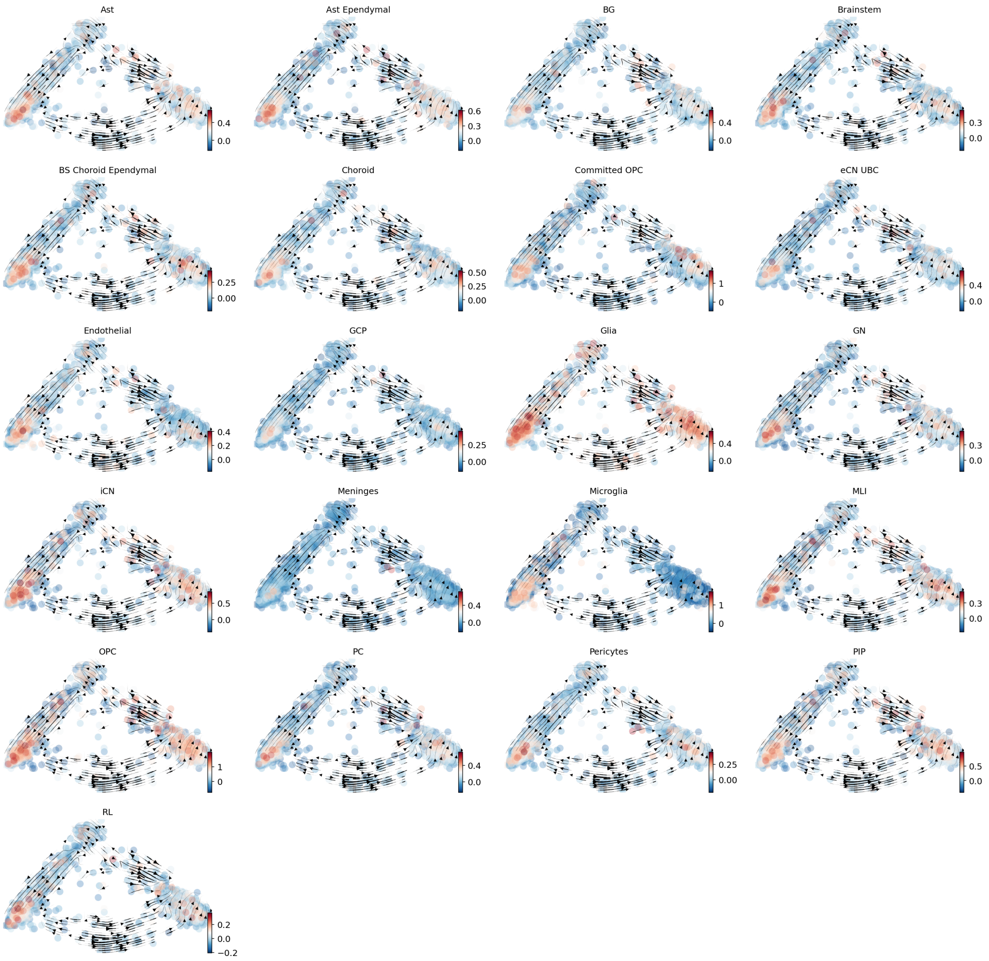

Fig. S14 Developmental signature module scores for human cerebellar cell-types for PA sample 1. The signatures for the cerebellar cell-types were determined based on the top markers for the annotated cell-types from Aldinger et al.

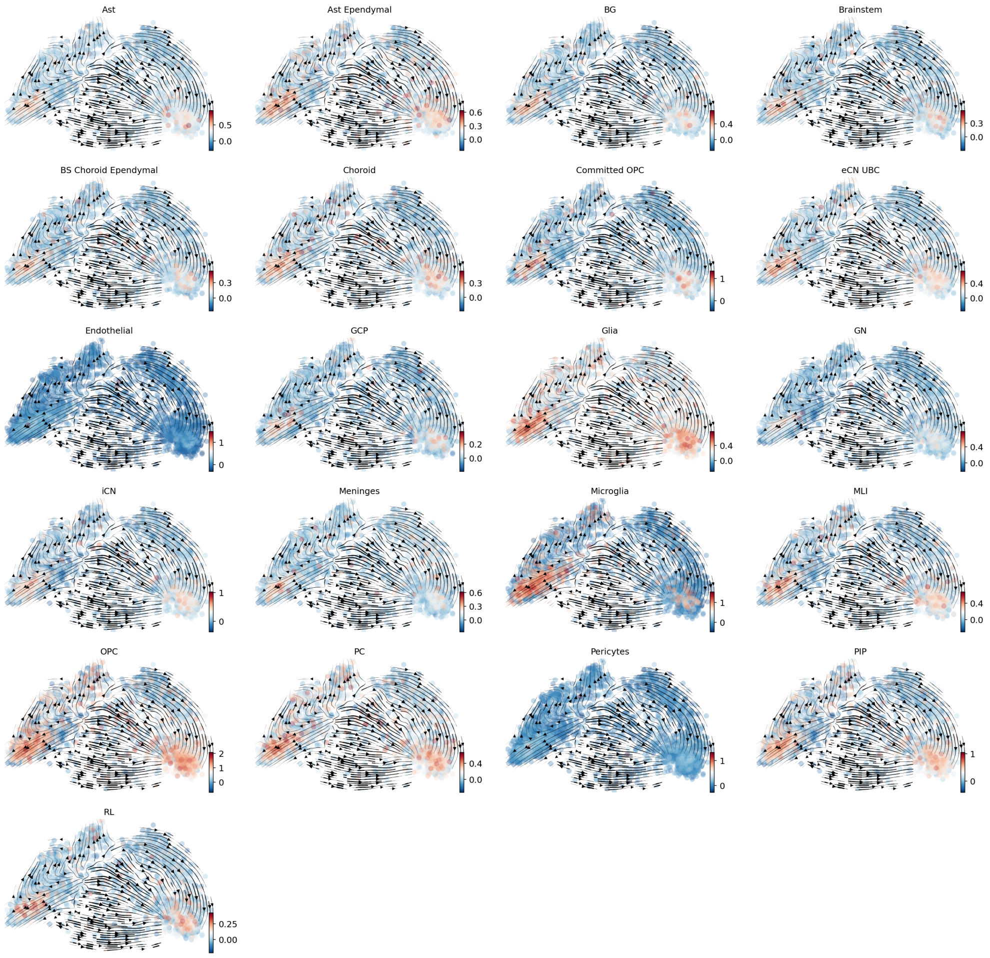

Fig. S15 Developmental signature module scores for human cerebellar cell-types for PA sample 2. The signatures for the cerebellar cell-types were determined based on the top markers for the annotated cell-types from Aldinger et al.

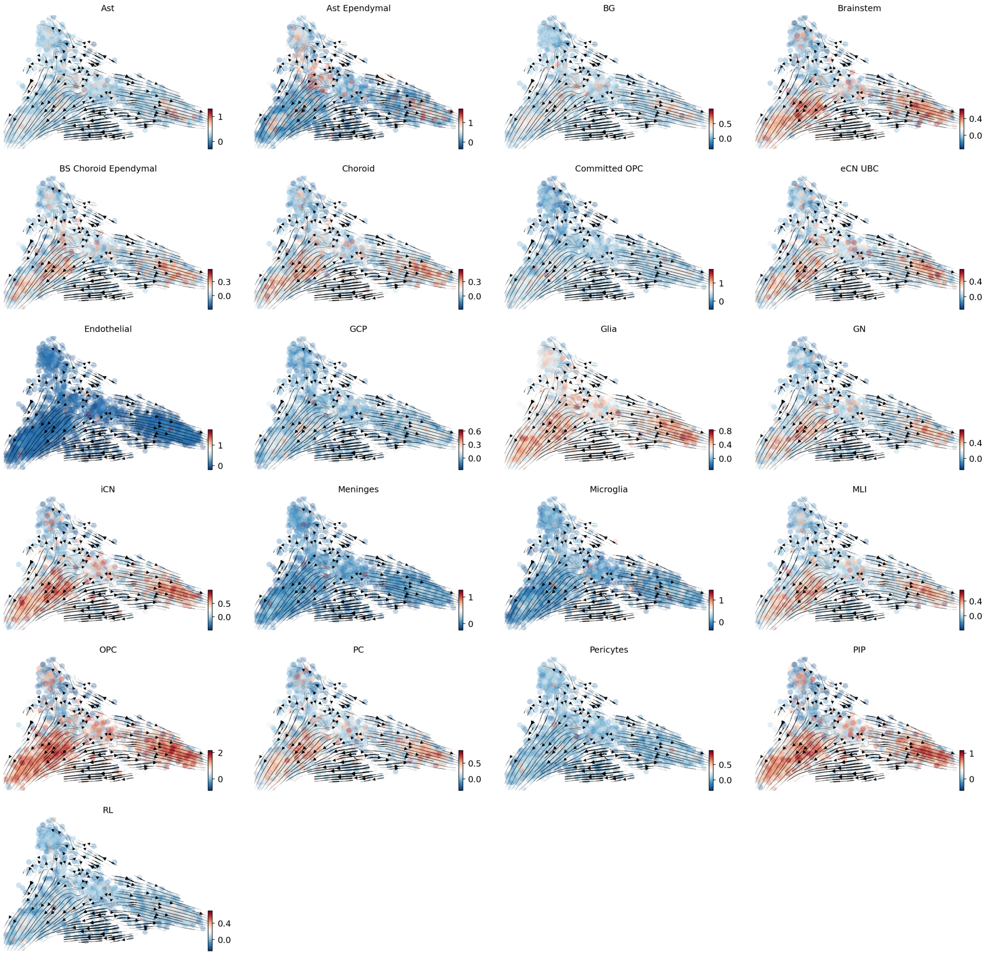

Fig. S16 Developmental signature module scores for human cerebellar cell-types for PA sample 3. The signatures for the cerebellar cell-types were determined based on the top markers for the annotated cell-types from Aldinger et al.

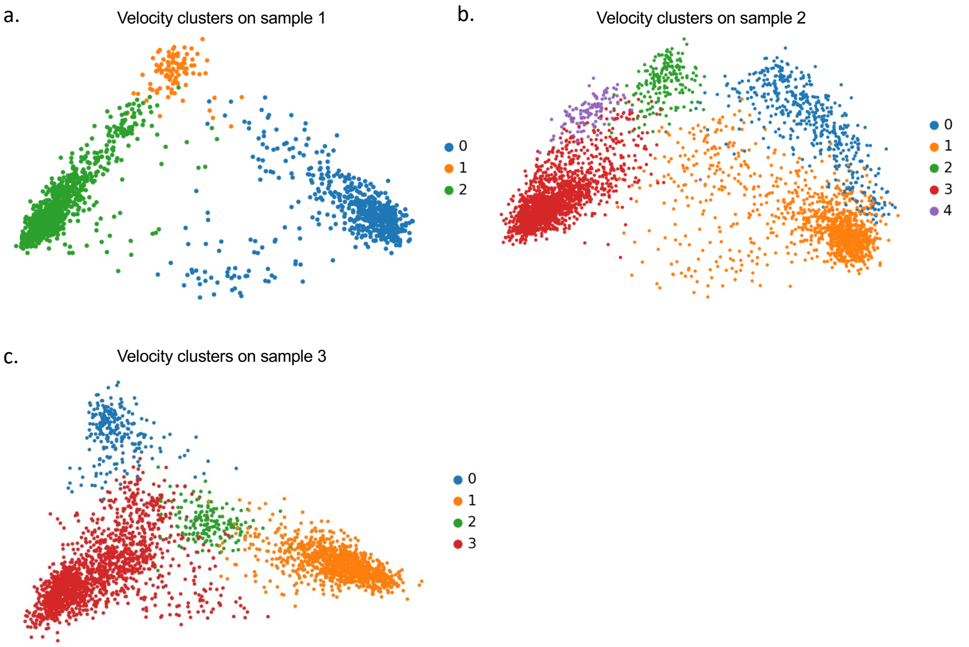

Fig. S17 Clusters on three PA samples. Clusters are obtained using Louvain clustering with resolution=0.05 on the DeepVelo velocity graph. The resulting clusters match the left-right branching pattern on the velocity plot on PCA coordinates (Result section 10). For the following driver gene selection steps per branch - On sample 1, we use cluster 2 as the left branch and cluster 0 as the right branch. On sample 2, we use clusters 2,3,4 as the left branch and clusters 0,1 as the right branch. On sample 3, we use cluster 3 as the left branch and cluster 1 as the right branch.

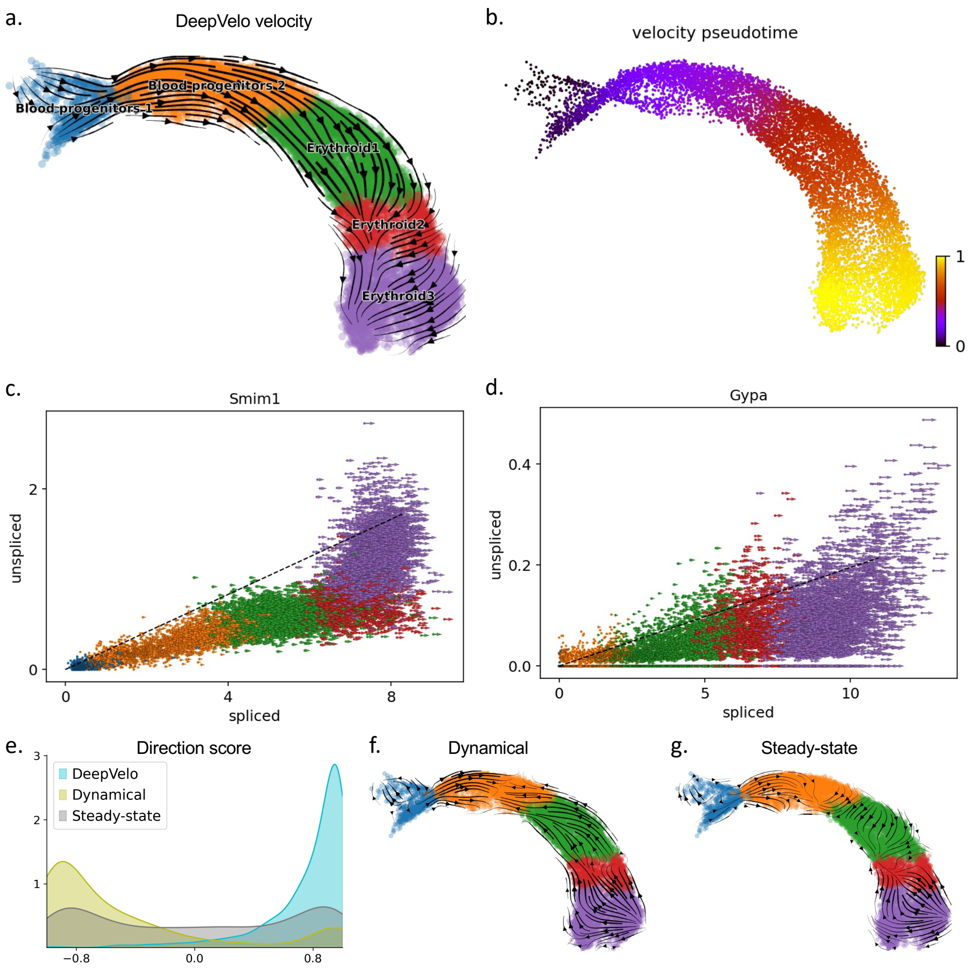

Fig. S18 DeepVelo RNA velocity estimate on the mouse gastrulation data. (a) Velocity vectors projected on the UMAP reduction of the data. (b) Diffusion pseudotime based on the velocity graph. (c,d) Velocity vectors projected onto the spliced and unspliced phase portraits of two multiple rate kinetic (MURK) genes. (e) Density plot of direction scores for the DeepVelo, dynamical, and steady-state models. (f,g) The velocity estimation by the scVelo dynamical model and the steady-state model.

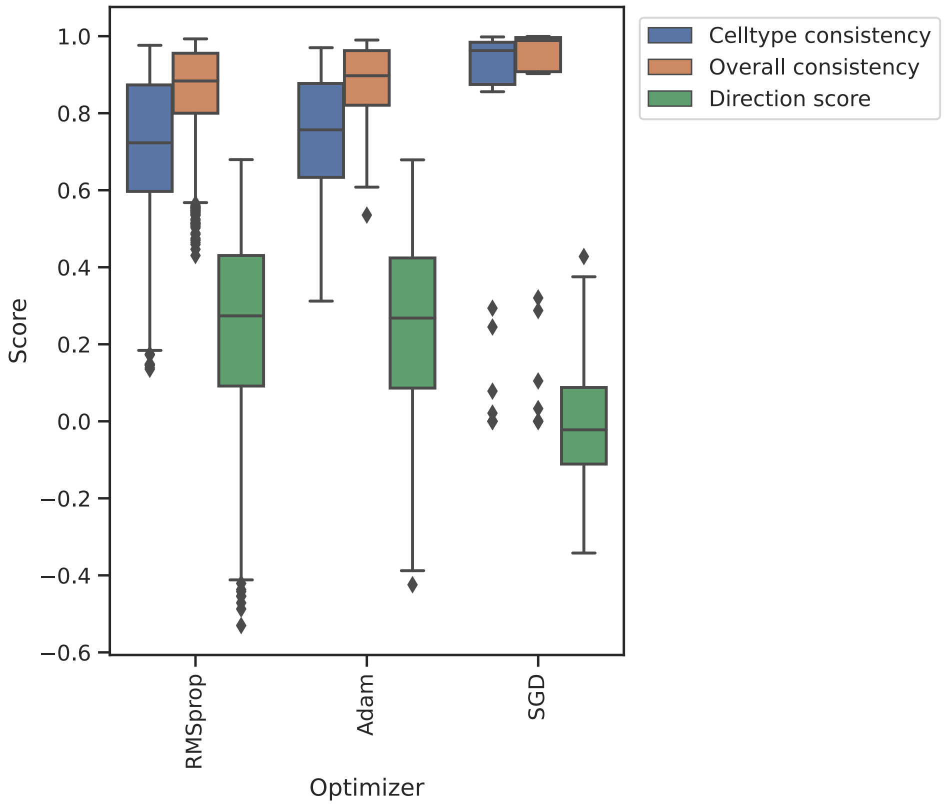

Fig. S19 Hyperparameter tuning results for optimizer type. Distribution of cell-type consistency, overall consistency, and direction scores for hyperparameter tuning run samples constrained to specific values for selected optimizer.

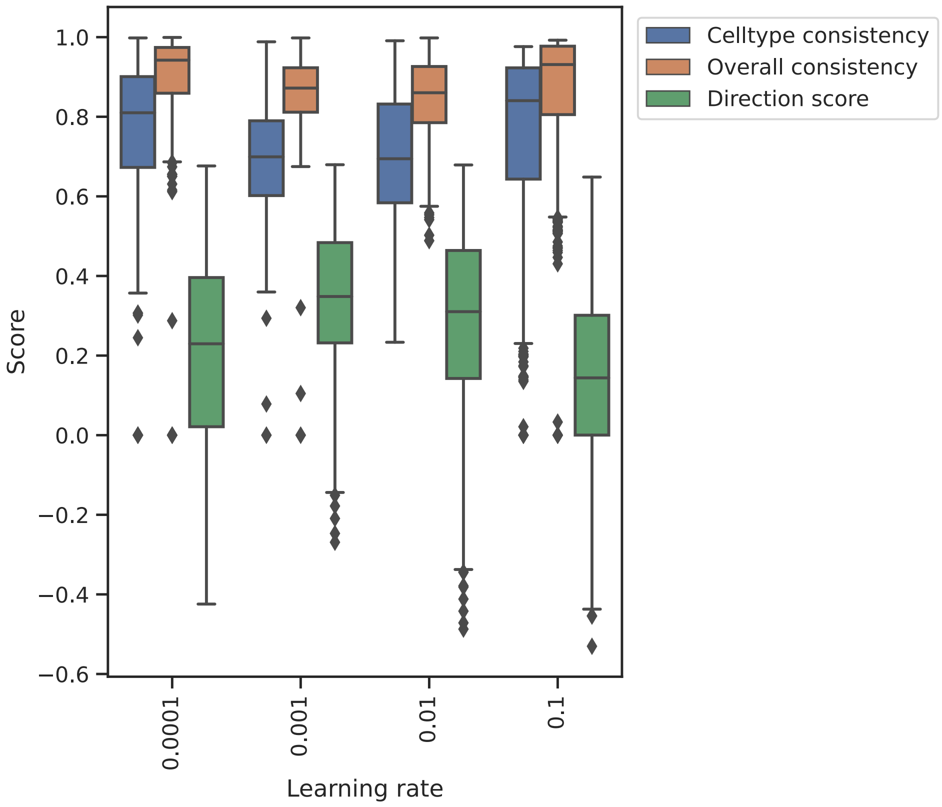

Fig. S20 Hyperparameter tuning results for optimization learning rate. Distribution of cell-type consistency, overall consistency, and direction scores for hyperparameter tuning run samples constrained to specific values for learning rate.

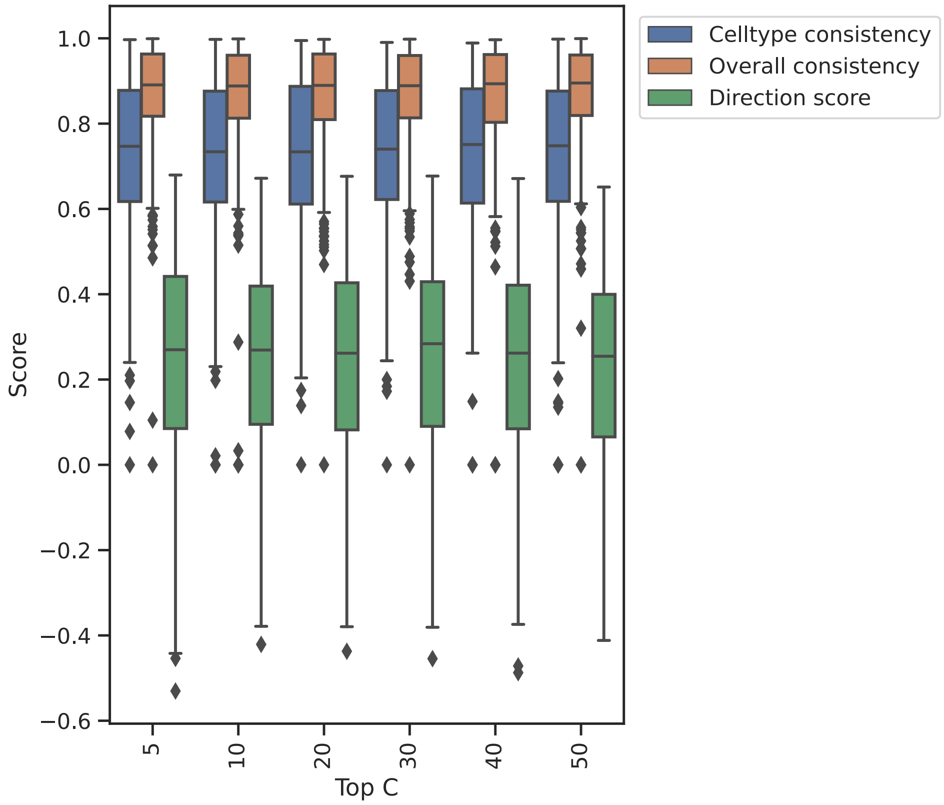

Fig. S21 Hyperparameter tuning results for the 'top C' hyperparameter. Distribution of cell-type consistency, overall consistency, and direction scores for hyperparameter tuning run samples constrained to specific values of 'top C'.

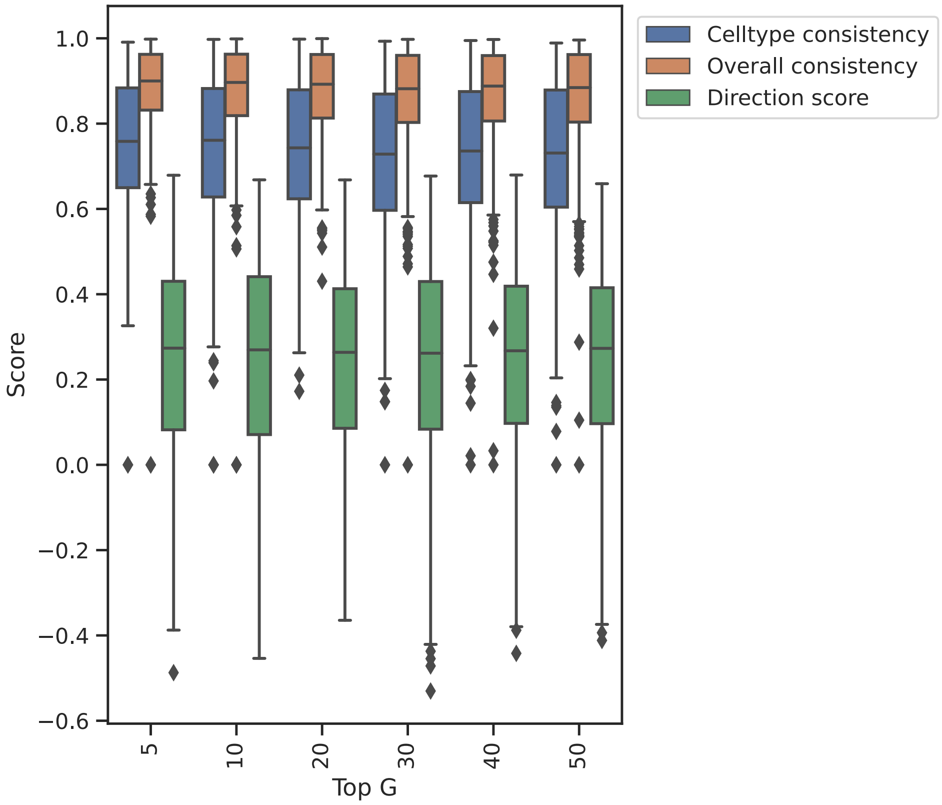

Fig. S22 Hyperparameter tuning results for the 'top G' hyperparameter. Distribution of cell-type consistency, overall consistency, and direction scores for hyperparameter tuning run samples constrained to specific values of 'top G'.

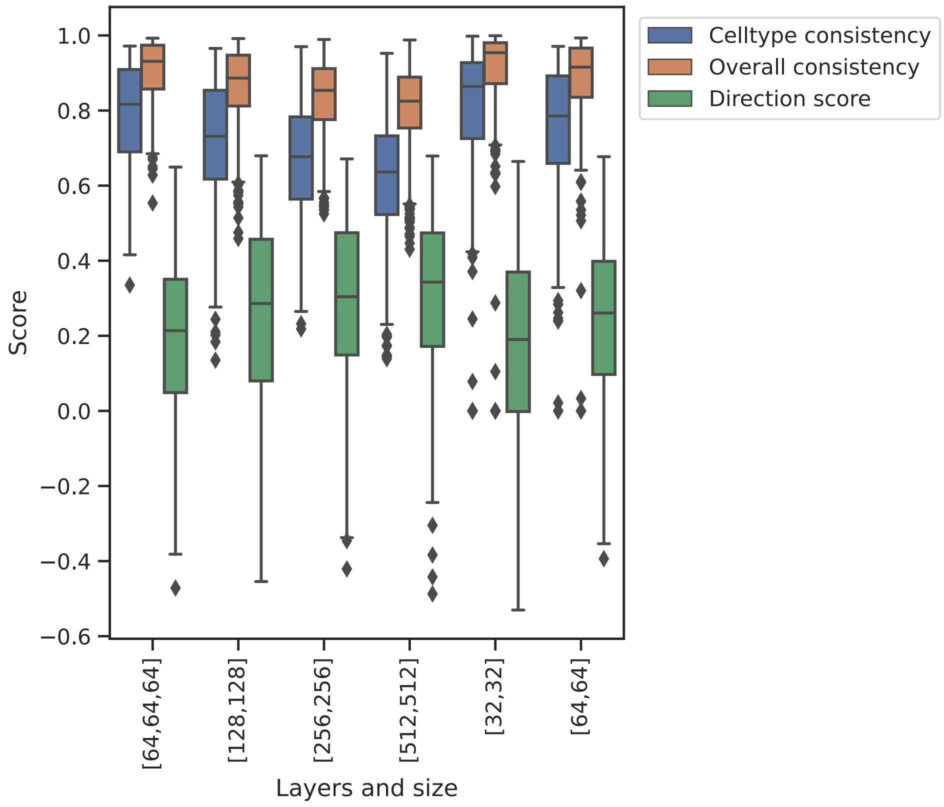

Fig. S23 Hyperparameter tuning results for the layers and size hyperparameter. Distribution of cell-type consistency, overall consistency, and direction scores for hyperparameter tuning run samples constrained to specific values of hidden layers and size in the DeepVelo network.

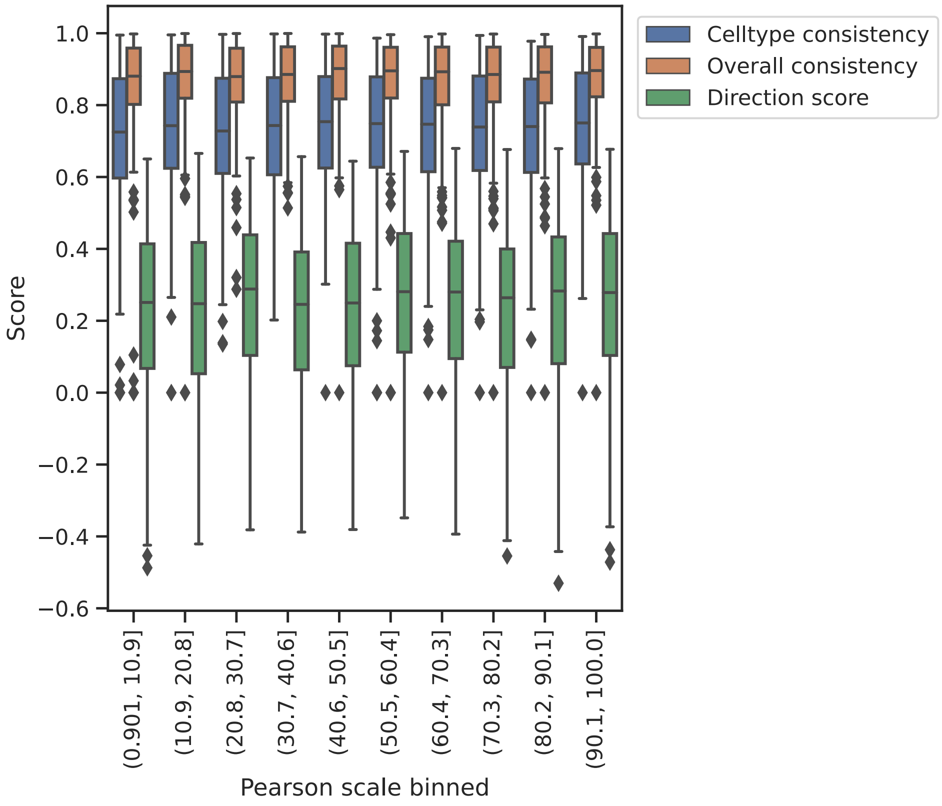

Fig. S24 Hyperparameter tuning results for the pearson coefficient scale hyperparameter. Distribution of cell-type consistency, overall consistency, and direction scores for hyperparameter tuning run samples constrained to specific values of pearson correlation coefficient scaling.

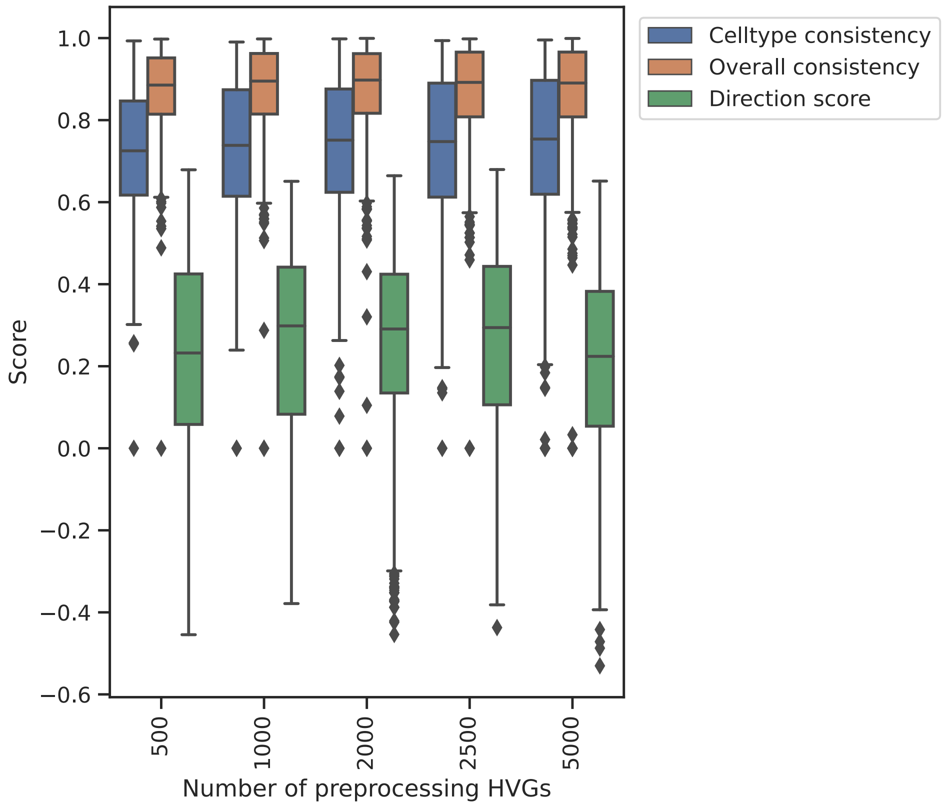

Fig. S25 Hyperparameter tuning results for the number of preprocessing highly-variable genes (HVGs). Distribution of cell-type consistency, overall consistency, and direction scores for hyperparameter tuning run samples constrained to specific values of preprocessing HVGs.

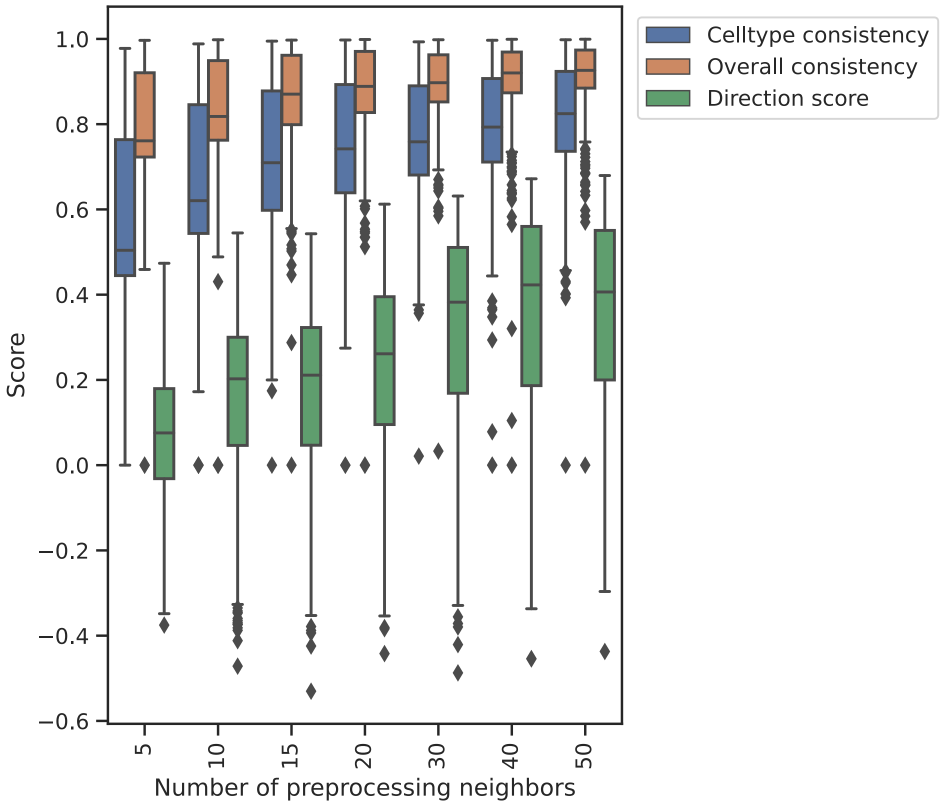

Fig. S26 Hyperparameter tuning results for the number of preprocessing neighbors for spliced/unspliced count smoothening. Distribution of cell-type consistency, overall consistency, and direction scores for hyperparameter tuning run samples constrained to specific values of the preprocessing number of neighbors.

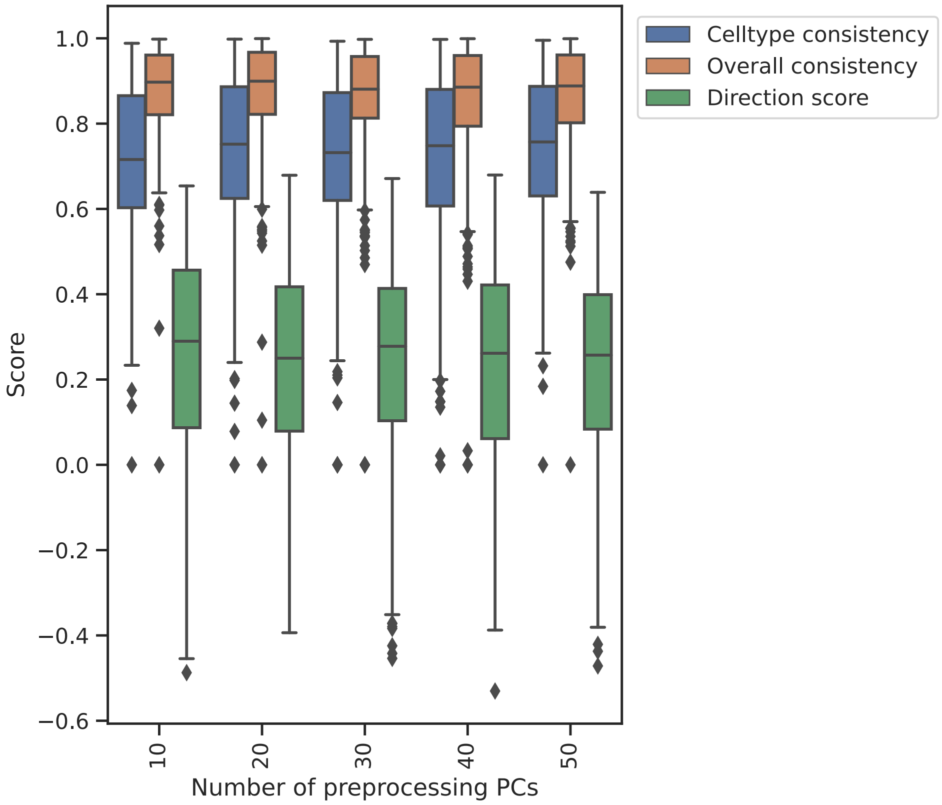

Fig. S27 Hyperparameter tuning results for the number of preprocessing principal components for input into the DeepVelo model. Distribution of cell-type consistency, overall consistency, and direction scores for hyperparameter tuning run samples constrained to specific values of the preprocessing number of principal components.

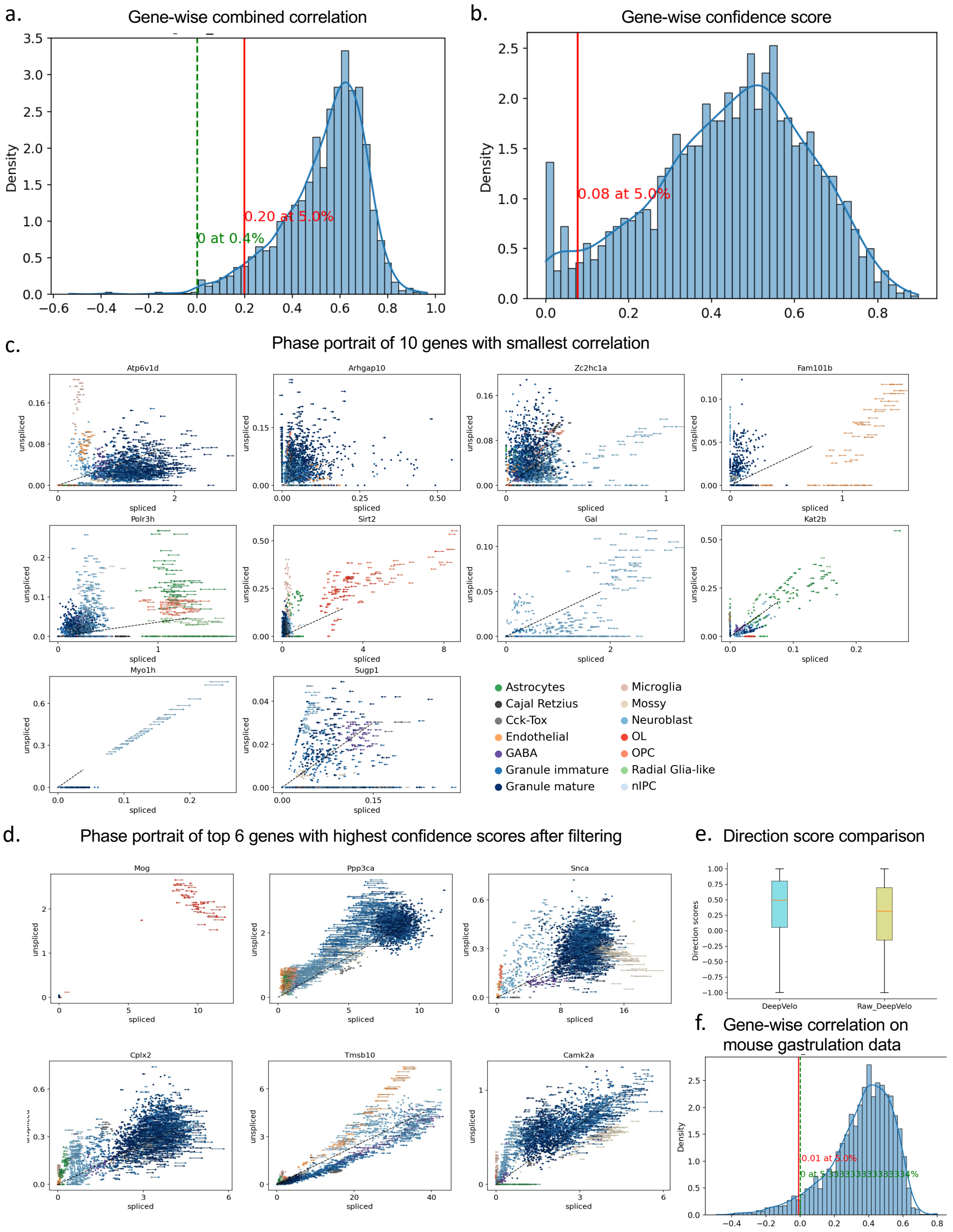

Fig. S28 Distribution of gene-wise confidence and correlation scores on the Dentate Gyrus dataset. (a,b) Gene-wise combined correlation and confidence scores. The red lines indicate the values at 5% percentile. The green line shows the percentile number at a correlation score of 0. The combined correlation score is the integration of the unspliced and spliced correlation terms exactly as in Eq.14. (c) Phase portraits of the 10 genes with the smallest correlation scores. (d) Phase portraits of top 6 genes with highest confidence scores after the filtering by confidence and continuity scores. (e) Comparison of direction scores. Using filtered genes (*DeepVelo*), DeepVelo demonstrates higher direction scores compared to the case when all genes are used (*Raw_DeepVelo*). (f) Gene-wise correlation scores on the mouse gastrulation data.

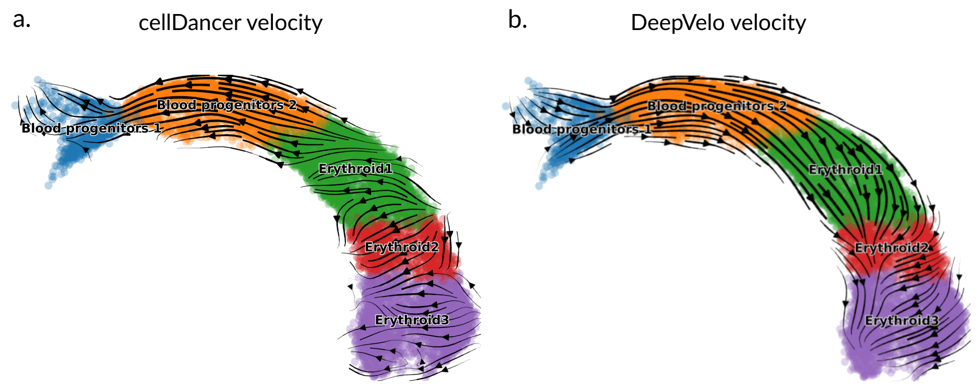

Fig. S29 Comparison of velocity estimates using the cellDancer (a) and DeepVelo (b) methods, for the mouse gastrulation data which is known to have genes with multi-faceted kinetics. In the cellDancer manuscript, the authors utilized a curated list of genes, that is not representative of a typical RNA velocity analysis. For a fair comparison that is representative, both methods were trained on and predicted the velocities for the same 2000 highly variable genes in mouse gastrulation data.
