# Supplementary material for: DeepVelo: deep learning extends RNA velocity to multi-lineage systems with cell-specific kinetics
Source: Genome Biol. 2024 Jan 19;25:27. doi: 10.1186/s13059-023-03148-9 (PMC10799431; doi:10.1186/s13059-023-03148-9)
Supplement: Supplementary file 11 — Additional file 11. Review history. [file 13059_2023_3148_MOESM11_ESM.docx]

Review History

This manuscript was reviewed elsewhere and the original reviewer reports and responses are not available.

The further revised manuscript submitted to Genome Biology was peer-reviewed and the comments by this reviewer are below.

**Reviewer 1**

**Comments to author:**

DeepVelo presents another choice for using RNA velocity to study dynamical processes. Regarding their responses to the reviewers' comments, although some responses are not perfect, in general they are OK. I just have two minor comments.

1. For the application of DeepVelo in rare cell types, the cell types mentioned by the authors such as nIPC and GABAergic interneurons should not be considered as rare cell types. They are common cell types in the brain. Although the authors did not test specifically on datasets containing "rare" cell types, they can discuss this point in the Discussion section by showing the robustness of the performance on the choice of the number of nearest neighbours (e.g., supplementary figure S21).

2. For the ability of DeepVelo to deal with datasets with batch effects/different time points, although the authors showed the distribution of time points in two datasets in supplementary figure S4, this figure was not referred and mentioned in the main text. The authors should clearly describe this in the main text to show the application of DeepVelo on datasets with varying time points.

**Authors Response**

**Point-by-point responses to the reviewers’ comments:**

Dear reviewer,

We want to thank you for taking the time to review our work. We greatly appreciate the constructive feedback you have provided, and believe that the manuscript has been improved as a result.

Regarding the two points raised, please find our responses outlined below:

R1. For the application of DeepVelo in rare cell types, the cell types mentioned by the authors such as nIPC and GABAergic interneurons should not be considered as rare cell types. They are common cell types in the brain. Although the authors did not test specifically on datasets containing "rare" cell types, they can discuss this point in the Discussion section by showing the robustness of the performance on the choice of the number of nearest neighbours (e.g., supplementary figure S21).

We thank the reviewer for raising this point regarding our responses. As per this concern, we have first emphasized the robustness of the DeepVelo model to the nearest neighbor calculation central to the continuity assumption in Results Section: DeepVelo is computationally robust and efficient across multiple scRNA-seq datasets. We have added the following point in that section:

Particularly important is the fact that DeepVelo is robust to the choice of number of neighbors to use for the future time point state calculation (Supplementary Figure S21). This calculation is central to the continuity assumption in DeepVelo (Methods - Continuity and Objectives), and this result highlights the robustness of continuity to hyperparameters central to the assumption.

We have elaborated on the aspect of rare cell-types and DeepVelo’s robustness to the number of neighbors in the continuity assumption in the Discussion section. We highlight the fact that, as the reviewer pointed out, although DeepVelo is robust to the number of neighbors in the hyperparameter experiments, this is not sufficient information to conclude that it can robustly perform velocity inference for rare cell-types. The potential challenge mainly comes from the rare cell groups that may have expression profiles considerably different from other cells, then it will be difficult to select their future neighbors out of the existing cell population. This may violate our continuity assumption.

We indicate that future studies should emphasize including cell-types that are less well characterized and less prevalent, and that this is an overall important direction for RNA velocity research:

The continuity assumption is central to the DeepVelo model, and although this assumption allows DeepVelo to be more expressive and less constrained, it does have its limitations. One potential limitation is the applicability of DeepVelo to cell-types that may be less prevalent or difficult to characterize, such as rare cell-types. Finding the correct future time point neighbors for these cell-types may be challenging, thus hampering the applicability of the continuity assumption. However, we do show that the DeepVelo model is robust to the choice of neighbors in the future time point state calculation. Further, DeepVelo works in a manner that is not biased by cell-type annotation, and as such, results will not be affected by incorrect annotation of rarer cell-types. Regardless, the challenge of modelling RNA velocity for rare cell-types is an important future research direction for RNA velocity methods, irrespective of their central assumptions, and future studies should emphasize the inclusion of data with less studied/rare cell-types.

R2. For the ability of DeepVelo to deal with datasets with batch effects/different time points, although the authors showed the distribution of time points in two datasets in supplementary figure S4, this figure was not referred and mentioned in the main text. The authors should clearly describe this in the main text to show the application of DeepVelo on datasets with varying time points.

We thank the reviewer for raising this point, and note that we did indeed miss referencing this figure and the general aspect of DeepVelo’s ability to deal with multiple timepoints and batches.

Regarding Supplementary Figure S4, we now reference this figure when describing the dentate gyrus neurogenesis and mouse hindbrain development datasets, and clearly indicate they are temporal in nature.

Further, at the end of the Results Section - Recovering complex transcriptional dynamics for individual cells using DeepVelo, we have added a short paragraph describing DeepVelo’s applicability to datasets with varying time points and batches:

Further, this result demonstrates DeepVelo's applicability to datasets which have multiple time points, and as such, may also contain batch effects. We further demonstrate DeepVelo's applicability in these scenarios in subsequent Results Sections involving multi-batch/temporal hindbrain and mesenchymal/chondrocyte organogenesis developmental datasets.
